# Supplementary material for: Loss of YhcB results in dysregulation of coordinated peptidoglycan, LPS and phospholipid synthesis during Escherichia coli cell growth
Source: PLoS Genet. 2021 Dec 23;17(12):e1009586. doi: 10.1371/journal.pgen.1009586 (PMC8741058; doi:10.1371/journal.pgen.1009586)
Supplement: S11 Table — (DOCX) [file pgen.1009586.s011.docx]

| **Strain or Library** | **Comments** | **References** |
| --- | --- | --- |
| BW25113 | Genotype: F- ∆(*araD-araB*)*567* ∆*lacZ4787*::rrnB-3 λ- *rph-1* ∆(*rhaD-rhaB*)*568* *hsdR514* | [1] |
| BW25113Δ*yhcB*::kan | *Keio isolate plate 69 Row E Column 10  Insertion: 22819_22820insCTGGGCGTG | [2] |
| BW25113Δ*yhcB*::kan | *Keio isolate plate 70 Row E Column 10 SNPs: 1085863C>T; 1972660C>T; 2910598G>C | [2] |
| BW25113::Tn*5*Cm | BW25113 mutagenized with a mini-Tn*5* carrying a chloramphenicol resistance selection marker | [3] |
| BW25113Δ*yhcB* | *yhcB* gene was first replaced with a kanamycin cassette via P1 transduction using a Keio library *yhcB* mutant as a donor strain. The cassette was then excised via recombination of the FRT sites leaving a 102 nt scar. | This study |
| BW25113::Tn*5*kan | BW25113 mutagenized with a mini-Tn*5* carrying a kanamycin resistance selection marker | This study |
| BW25113Δ*yhcB*::Tn*5*kan | BW25113Δ*yhcB* mutagenized with a mini-Tn*5* carrying a kanamycin resistance selection marker | This study |
| BW25113Δ*dacA*::kan |  | This study |
| BW25113Δ*lpxM*::kan |  | This study |
| BW25113Δ*mepS*::kan |  | This study |
| BW25113Δ*wecF*::kan |  | This study |
| BW25113Δ*amiD*::kan |  | This study |
| BW25113Δ*yhcB*Δ*amiD*::kan |  | This study |
| BW25113Δ*mltG*::kan |  | This study |
| BW25113Δ*yhcB*Δ*mltG*::kan |  | This study |
| BW25113Δ*mlaD*::kan |  | This study |
| BW25113Δ*yhcB*Δ*mlaD*::kan |  | This study |
| BW25113Δ*nlpI*::kan |  | This study |
| BW25113Δ*yhcB*Δ*nlpI*::kan |  | This study |
| BW25113Δ*fabF*::kan |  | This study |
| BW25113Δ*yhcB*Δ*fabF*::kan |  | This study |
| BW25113Δ*yhcB*Δ*ftsH*::kan |  | This study |

**Table S11. Strains used in this study**

*please note, we have not sequenced other Keio libraries.

**References**

1. Datsenko KA, Wanner BL. One-step inactivation of chromosomal genes in *Escherichia coli* K-12 using PCR products. Proc Natl Acad Sci U S A. 2000;97: 6640–5. doi:10.1073/pnas.120163297

2. Baba T, Ara T, Hasegawa M, Takai Y, Okumura Y, Baba M, et al. Construction of *Escherichia coli* K-12 in-frame, single-gene knockout mutants: the Keio collection. Mol Syst Biol. 2006;2: 2006.0008. doi:10.1038/msb4100050

3. Goodall ECA, Robinson A, Johnston IG, Jabbari S, Turner KA, Cunningham AF, et al. The Essential Genome of *Escherichia coli* K-12. Chen SL, editor. MBio. 2018;9: e02096-17. doi:10.1128/mBio.02096-17
