# Supplementary material for: Loss of YhcB results in dysregulation of coordinated peptidoglycan, LPS and phospholipid synthesis during Escherichia coli cell growth
Source: PLoS Genet. 2021 Dec 23;17(12):e1009586. doi: 10.1371/journal.pgen.1009586 (PMC8741058; doi:10.1371/journal.pgen.1009586)
Supplement: S12 Table — (DOCX) [file pgen.1009586.s012.docx]

| **Plasmid** | **Comments** | **References** |
| --- | --- | --- |
| pCP20 | λ*cI857*(ts) *repA101*(ts) *oriR101 bla cat* λpR‐FLP | [1] |
| pKD4 | *oriRγR6k* *bla* FRT::kan::FRT | [1] |
| pBAD/*Myc*-His A | *oripBR322* Amp^R^ | Invitrogen |
| pBAD-*yhcB* | Expression of *yhcB* under arabinose inducible promoter. Stop codon of *yhcB* included. | This study |
| pBAD-*yhcB*ΔTM | YhcB_21‑132_ | This study |
| pBAD-*yhcB*ΔPRDY | YhcB_1‑99_ | This study |
| pBAD-*yhcB*ΔNPF | YhcB_1‑90_ | This study |
| pTrc99A-*lpxC* | *lpxC* derived from MG1655 cloned between Kpn*I* and Hind*III* under the control of a trc promoter. *oripBR322* Amp^R^ | Gift from Yaoqin Hong |
| pASK-IBA2C | *oriColE1* Tet inducible Cm^R^ | IBA Life Sciences |
| pASK-IBA2C-*uppS* | *uppS (ispU)* derived from BW25113 cloned between the Xba*I* and Hind*III* restriction sites, under the control of anhydrous tetracycline inducible promoter | This study |
| pASK-IBA2C-*cdsA* | *cdsA* derived from BW25113 cloned between the Xba*I* and Hind*III* restriction sites, under the control of anhydrous tetracycline inducible promoter | This study |
| pACYCDuet-1 | *oriP15A lacI* Cm^R^, | Novagem |
| pACYCDuet-1-*cdsA*_AS_ | Antisense *cdsA*, derived from BW25113, under the control of T7 promoter. Cm^R^ | This study |

**Table S12. Plasmids used in this study**

**References**

1. Datsenko KA, Wanner BL. One-step inactivation of chromosomal genes in *Escherichia coli* K-12 using PCR products. Proc Natl Acad Sci U S A. 2000;97: 6640–5. doi:10.1073/pnas.120163297
