# Supplementary material for: Loss of YhcB results in dysregulation of coordinated peptidoglycan, LPS and phospholipid synthesis during Escherichia coli cell growth
Source: PLoS Genet. 2021 Dec 23;17(12):e1009586. doi: 10.1371/journal.pgen.1009586 (PMC8741058; doi:10.1371/journal.pgen.1009586)
Supplement: S13 Table — (DOCX) [file pgen.1009586.s013.docx]

| **Primer name** | **Sequence (5′-3′)** |
| --- | --- |
| yhcB_check.F | TAAGCGCCTTCAGGTATTGC |
| yhcB_check.R | CCGACACTTAACGCTAATGC |
| pBAD_ins_check.F | GCTATGCCATAGCATTTTTATCC |
| pBAD_ins_check.R | CGTTCTGATTTAATCTGTATCAGG |
| rmTM_yhcB_pBAD.F | ATGCGTTTTGGTAATCGTAAAC |
| rmTM_yhcB_pBAD.R | GGTTAATTCCTCCTGTTAGCC |
| STOP_yhcB_pBAD.F | TAAGCTTGGGCCCGAAC |
| rm_PRDY_yhcB_pBAD.R | CAGACGATTACGGAACGG |
| rm_NPF_yhcB_pBAD.R | AGCAGACAGTTCCGGCAG |
| ftsH_KO.F | AACACAGTTGTAATAAGAGGTTAATCCCTTGAGTGACATGGTGTAGGCTGGAGCTGCTTC |
| ftsH_KO.R | CATCTGATGCGGGAACTTACTTGTCGCCTAACTGCTCTGACATATGAATATCCTCCTTAG |
| ftsH_check.F | TTCGTAAGCCGGACTCTTCTC |
| ftsH_check.R | CAGCGAGTTATGCGTGCCAC |
| Kan_int.R | CATGCTCTTCGTGCAGATCA |
| ispU_XbaI_RBS_F | AATCTAGATAACGAGGAAAACGCGTGATGTTGTCTGCTACTC |
| ispU_HindIII_R | AAAAGCTTTCAGGCTGTTTCATCACCGG |
| cdsA_XbaI_RBS_F | AATCTAGATAACGAGGAAAACGCTTGCTGAAGTATCGCCTGATATC |
| cdsA_HindIII_R | AAAAGCTTTTAAAGCGTCCTGAATACCAGTAAC |
| pACYC-Duet_GB_F | GAGCAATAACTAGCATAACC |
| pACYC-Duet_GB_R | AAGTTAAACAAAATTATTTCTACAGGG |
| cdsAas_pACYCDuet_GB_F | CCCTGTAGAAATAATTTTGTTTAACTTTTAAAGCGTCCTGAATACCAG |
| cdsAas_pACYCDuet_GB_R | GTTATGCTAGTTATTGCTCTTGCTGAAGTATCGCCTGATATC |

**Table S13. Oligonucleotides used in this study**
