# Supplementary figures and images for: Loss of YhcB results in dysregulation of coordinated peptidoglycan, LPS and phospholipid synthesis during Escherichia coli cell growth
Source: PLoS Genet. 2021 Dec 23;17(12):e1009586. doi: 10.1371/journal.pgen.1009586 (PMC8741058; doi:10.1371/journal.pgen.1009586)

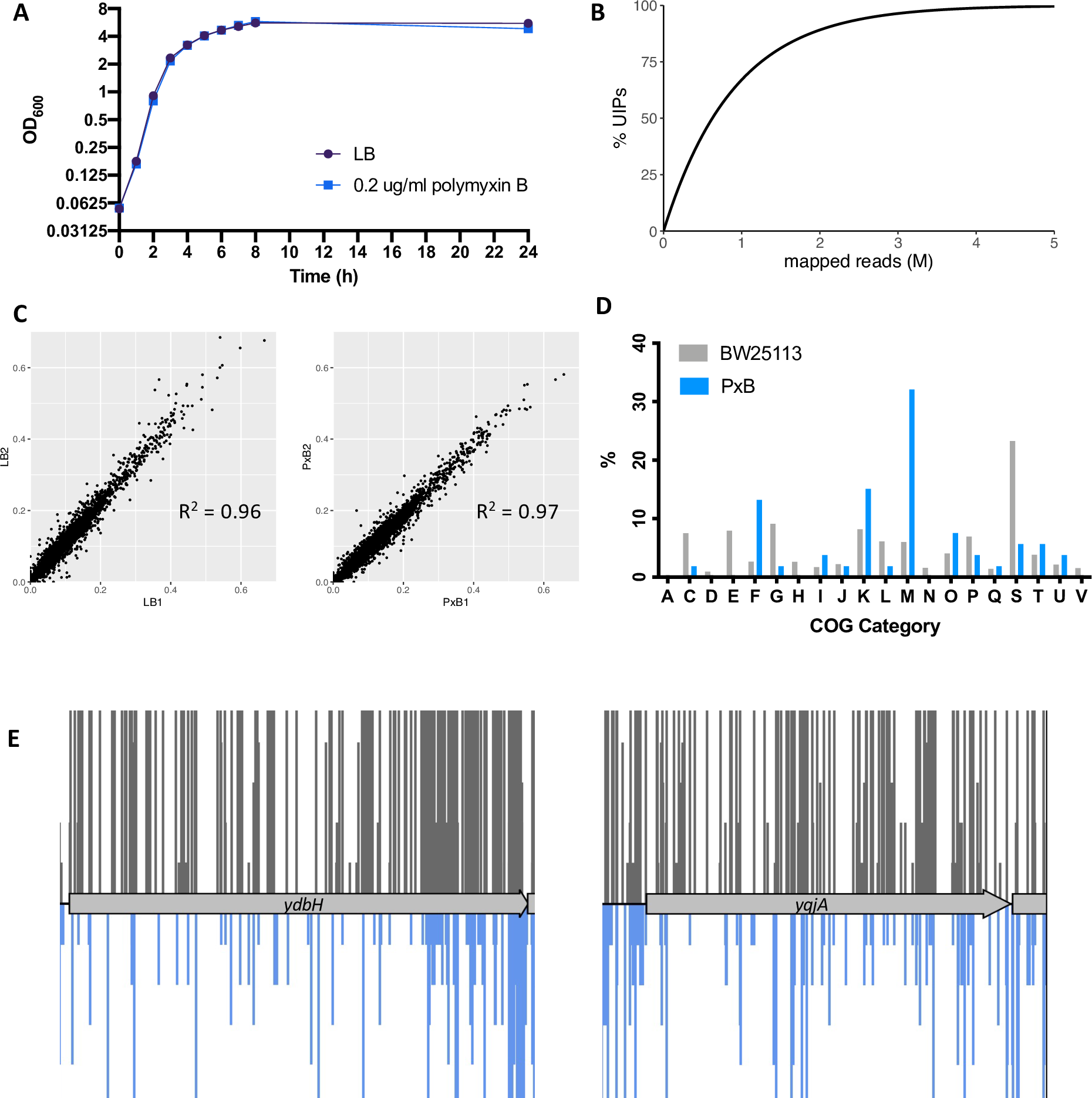

Supplement: S1 Fig — (A) 0.2 μg/ml of polymyxin B in 50 ml LB does not inhibit growth of E. coli BW25113, under the conditions used to screen the library. One representative growth curve is shown, consistent with 5 repeats. (B) Calculation of the number of mapped reads needed to sample a given percentage of the transposon library. The equation I=s−s(s−1s)n was used to estimate the number of mapped reads required to sample a given proportion of the dataset. s = 1,000,000 was taken as the total number of possible mutants (total transposon insertion sites). I = insertions identified, n = number of mapped reads. This data was used to calculate the approximate percentage of unique insertions identified for a given number of mapped reads, for a library of 1,000,000 unique mutants. (C) Comparison of insertion index scores (a measure of insertion density) per gene for each replicate of the library either exposed to LB only, or LB supplemented with polymyxin B. The scatter plots show the correlation coefficient for insertion density of each gene between replicates. (D) The relative abundance of Cluster of Orthologous Groups (COG) categories for all genes of BW25113 (grey) and for the 54 genes identified as required for growth in sub-inhibitory concentrations of polymyxin B (PxB; blue). (E) The transposon insertion profiles of yqjA and ydbH following outgrowth in LB only (grey, above) or in LB supplemented with polymyxin B (blue, below). The transposon insertion position along each gene is marked by a vertical line.The vertical line size corresponds with read depth, with visibly fewer transposon-insertion sites identified within ydbH and yqjA following outgrowth in polymyxin B. (TIF) [file pgen.1009586.s014.tif]

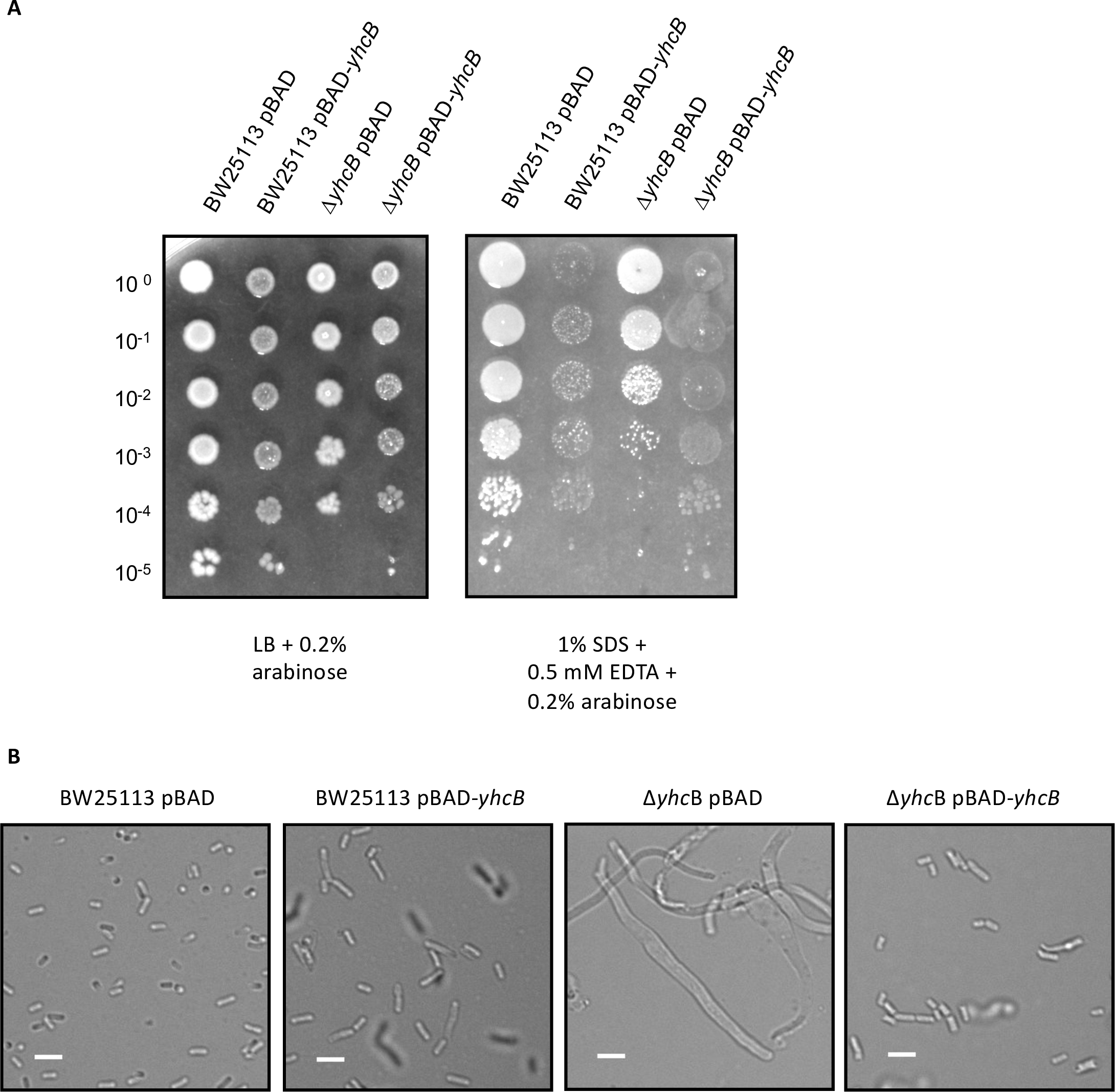

Supplement: S2 Fig — (A) 10-fold serial-dilution of overnight cultures grown in LB, inoculated onto LB agar plates supplemented with 0.2% arabinose, with and without 1% SDS + 0.5 mM EDTA. Strains are carrying a pBad-Myc-His-A with or without the yhcB CDS under the control of an arabinose promoter. (B) DIC microscopy images of day cultures grown in LB supplemented with 0.4% arabinose and 100 μg/ml carbenicillin (to maintain plasmids), with a 5 μm scale bar for reference. (TIF) [file pgen.1009586.s015.tif]

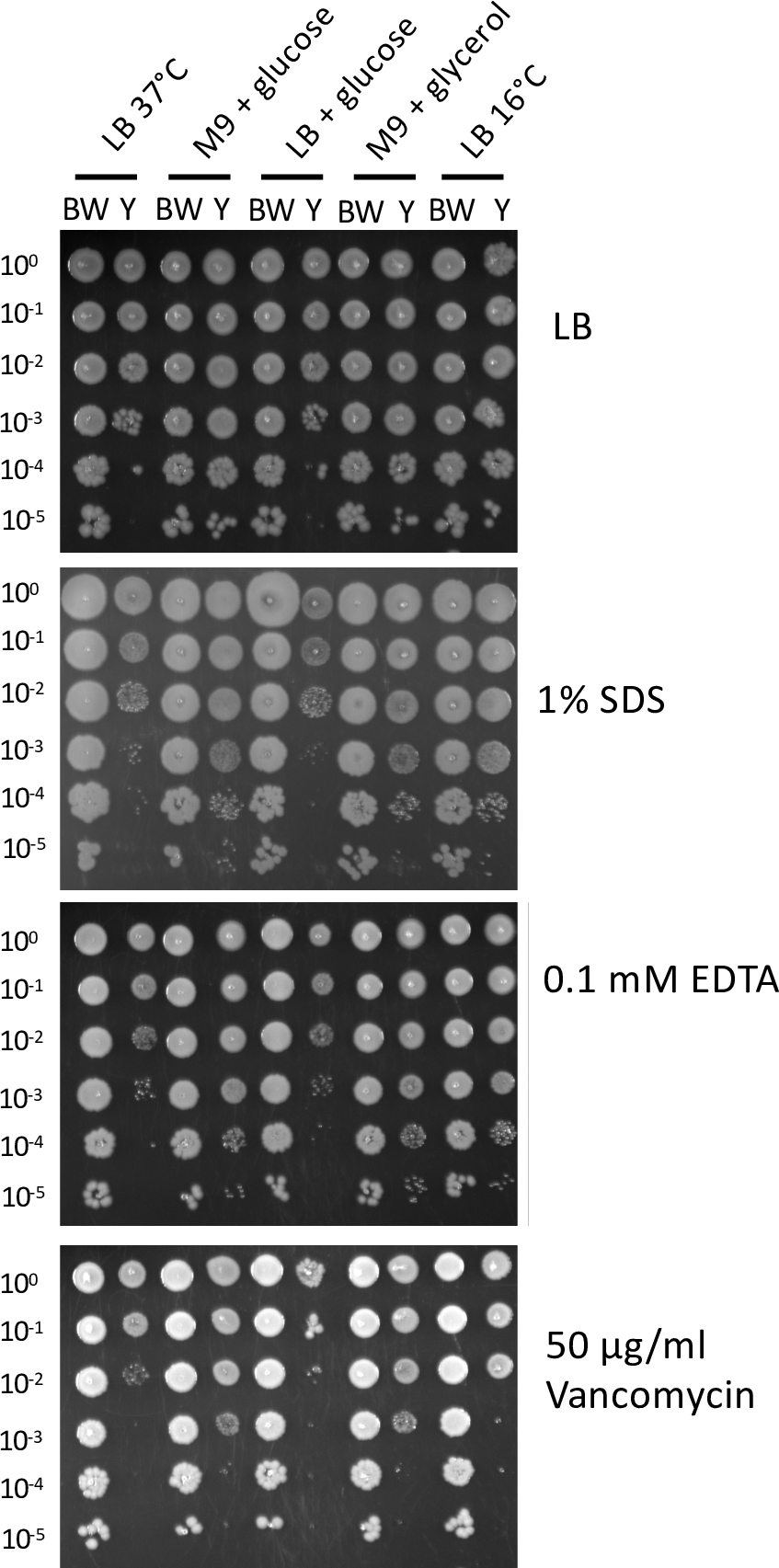

Supplement: S3 Fig — 10-fold serial-dilution of overnight cultures of the parent strain E. coli BW25113 (BW) and ΔyhcB strain (Y) grown to late stationary phase under different conditions (LB at 37°C; M9 + 0.4% glucose at 37°C; LB + 0.4% glucose at 37°C; M9 + 0.4% glycerol at 37°C; LB at 16°C) normalized to an OD600 of 1.00 and inoculated onto LB agar plates supplemented with and without 1% SDS; 0.1 mM EDTA or 50 μg/ml vancomycin. (TIF) [file pgen.1009586.s016.tif]

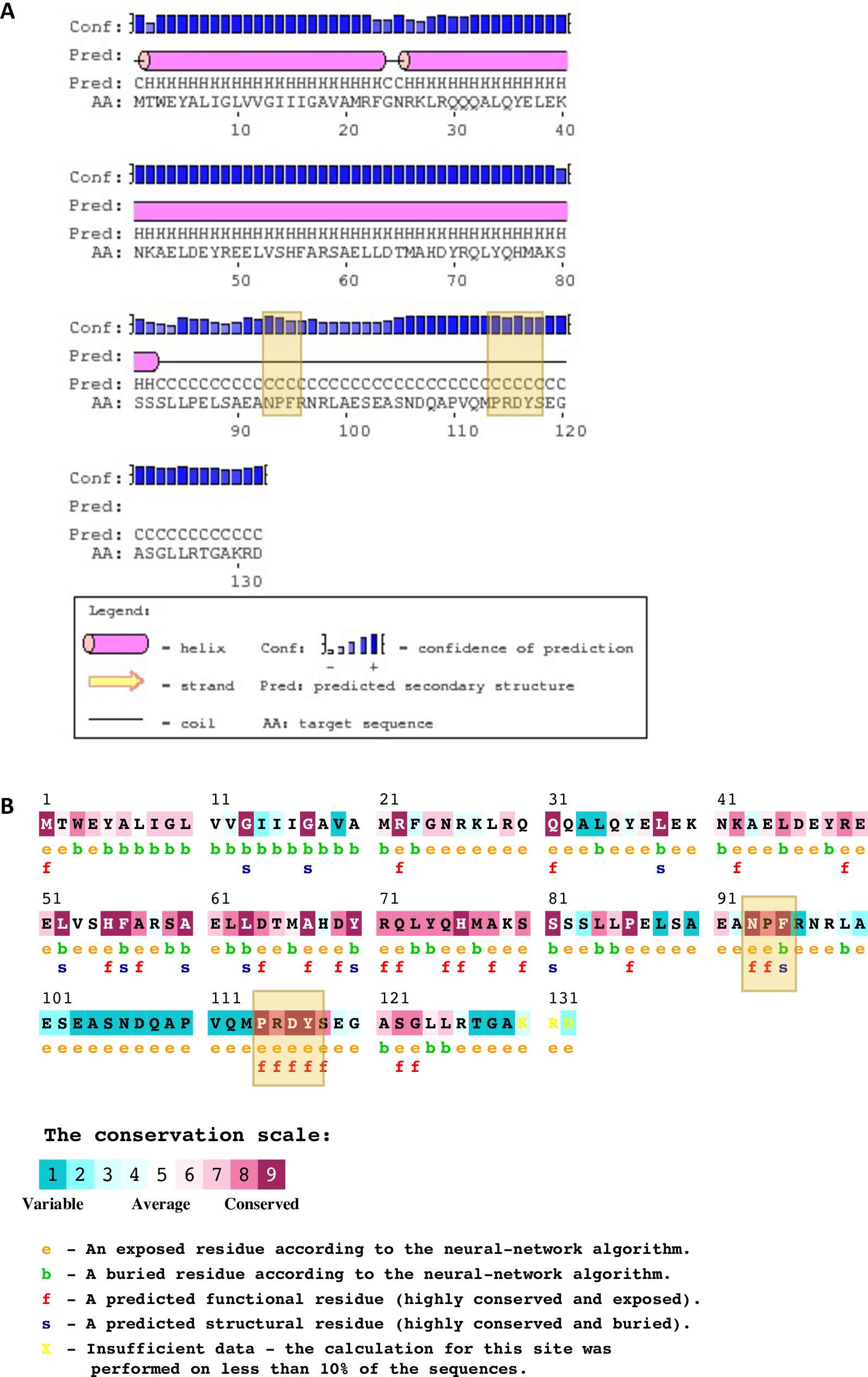

Supplement: S4 Fig — (A) PSIPRED secondary structure prediction of YhcB. (B) Conserved residues of YhcB predicted by ConSurf. Conserved ‘NPF’ and ‘PRDY’ motifs are highlighted with amber boxes in both panels. (TIF) [file pgen.1009586.s017.tif]

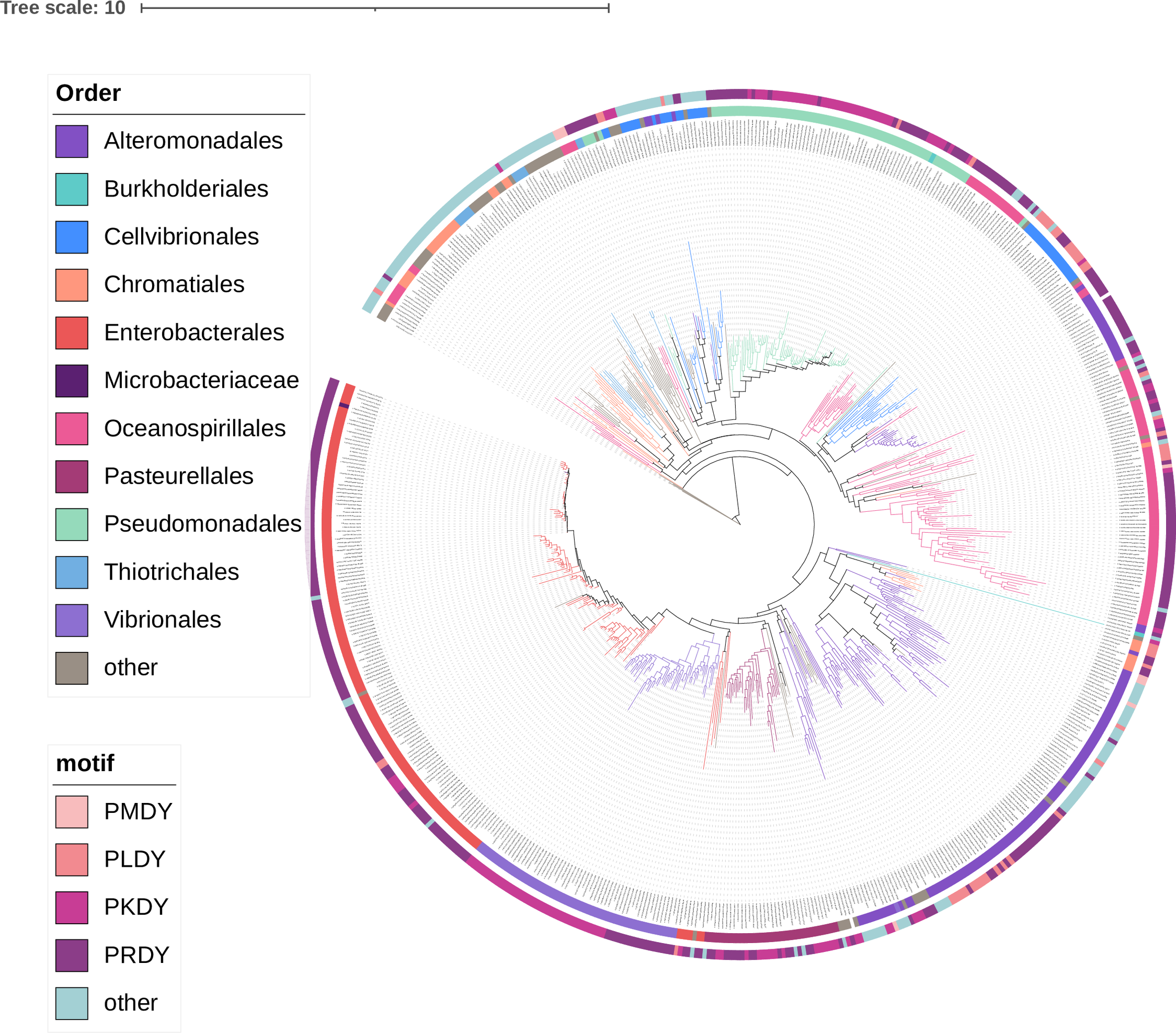

Supplement: S5 Fig — Phylogenetic analysis displaying conservation of YhcB in bacterial reference genomes. Branches of the tree, and second outermost track, are coloured according to taxonomic Order. The outermost track is coloured according to the amino acid residues within the ‘PXDY’ domain conserved among species. The label ‘other’ represents those that had a different sequence to the four listed. (TIF) [file pgen.1009586.s018.tif]

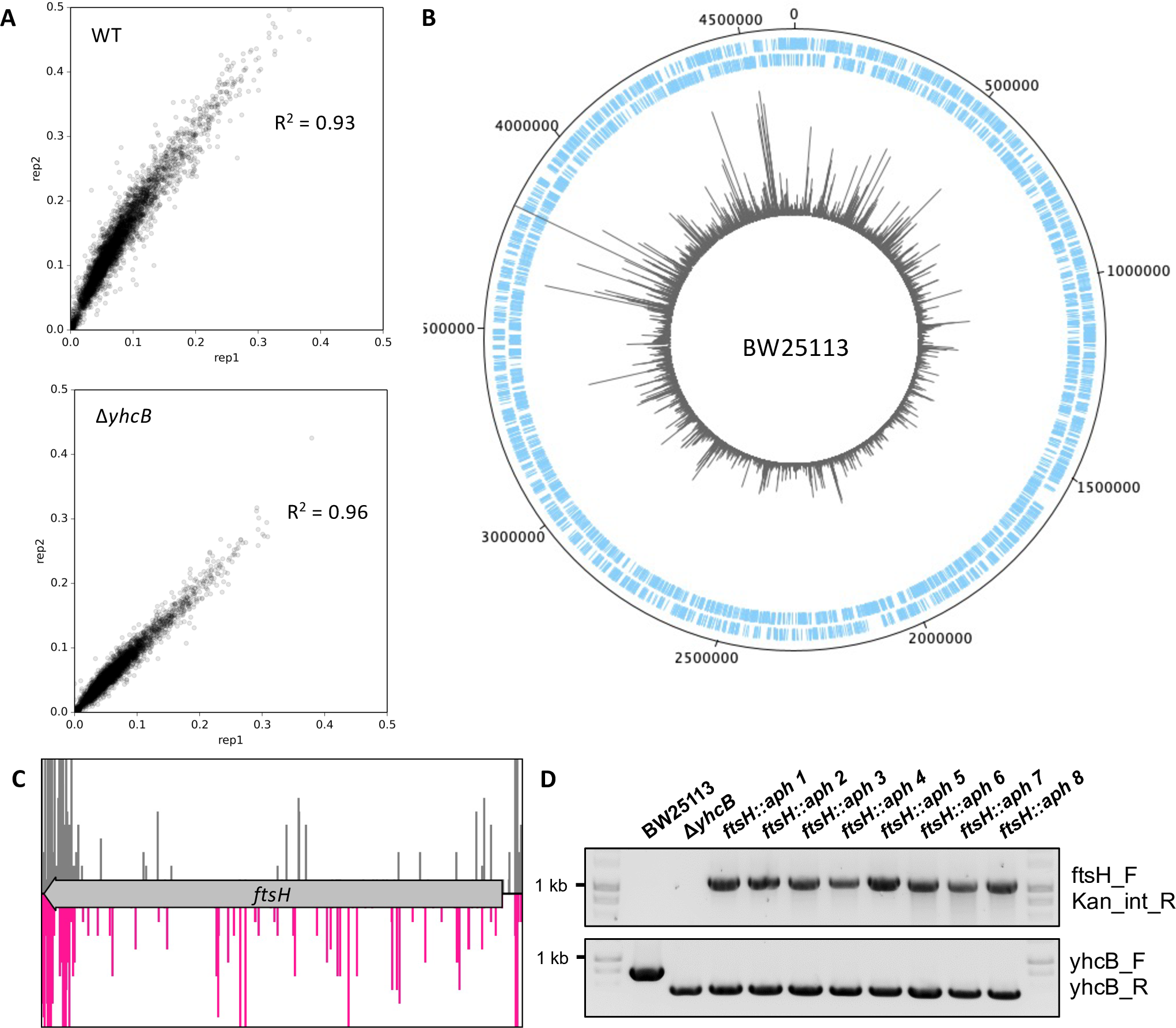

Supplement: S6 Fig — (A) Comparison of insertion index scores (IIS) between technical replicates of each library. (B) The BW25113 transposon library. A genome map of BW25113 starting at the annotation origin, with the sense and antisense coding sequences of BW25113 shown in blue, respectively, and the position and frequency of sequenced transposon insertion events shown in grey. (C) The transposon insertion profile of ftsH shown for both libraries. Peaks represent the abundance of detected transposon insertion events for each library: wild-type (grey, above) and yhcB (pink, below) with read frequency capped at 10. (D) PCR amplification of the yhcB and ftsH loci to confirm yhcB-deletion and ftsH-replacement with a kanamycin resistance cassette in these strains. (TIF) [file pgen.1009586.s019.tif]

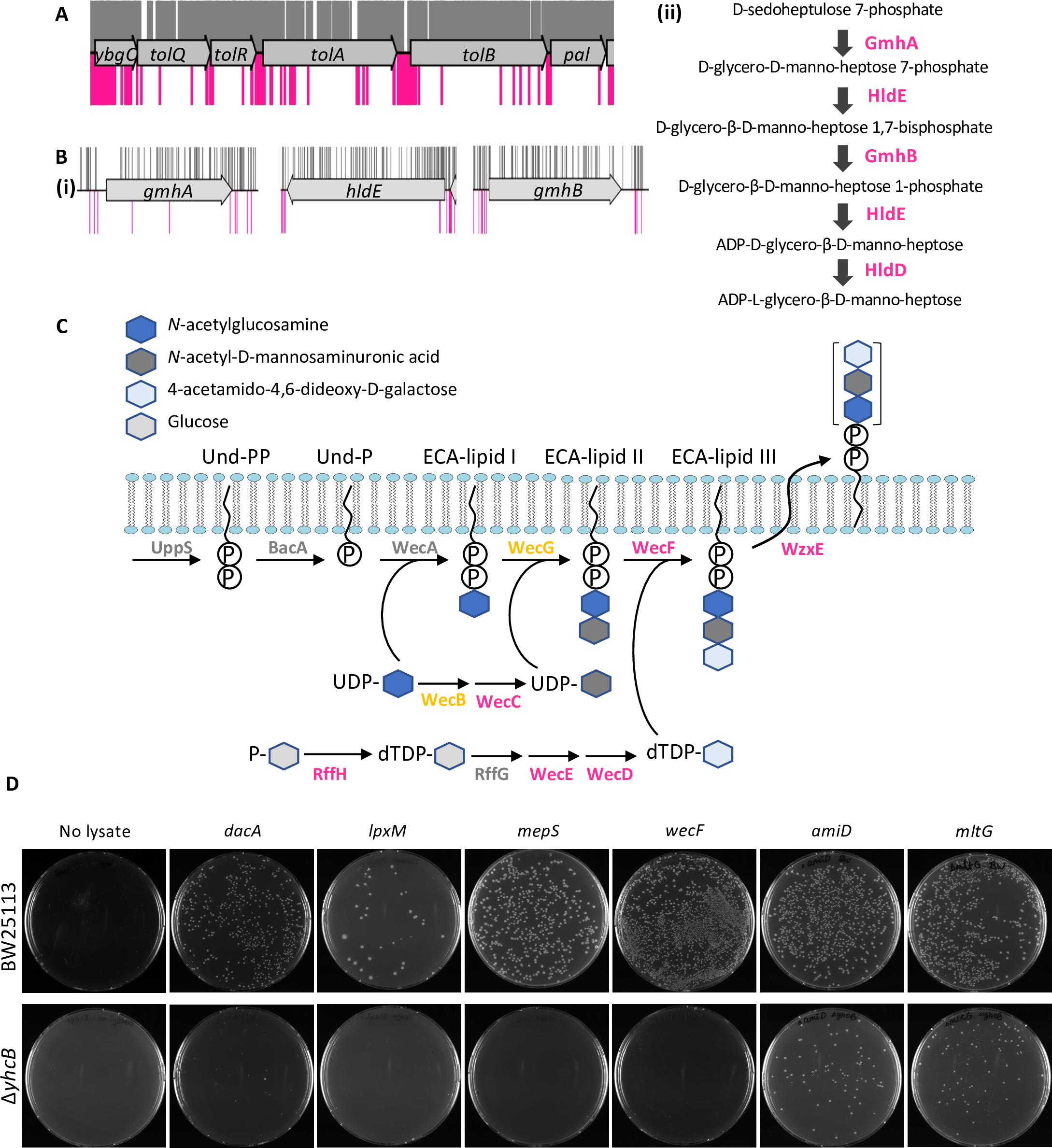

Supplement: S7 Fig — Transposon insertion data of the yhcB library shown in pink, below, control library shown in grey, above, the gene track with all insertion data capped at a frequency of 1. (A) Transposon insertion profile of the tol-pal operon. (B) The heptose biosynthetic pathway, required for LPS core biosynthesis. (C) Schematic of the ECA biosynthesis pathway adapted from Jorgenson et al. [80]. (D) Representative LB agar plates of colonies recovered following P1 transduction, representative of n = 2 experiments. Abbreviations: Enterobacterial Common Antigen (ECA); Undecaprenyl phosphate (Und-P); Phosphate (P); Adenosine diphosphate (ADP); deoxythymidine diphosphate (dTDP); Uridine diphosphate (UDP). (TIF) [file pgen.1009586.s020.tif]

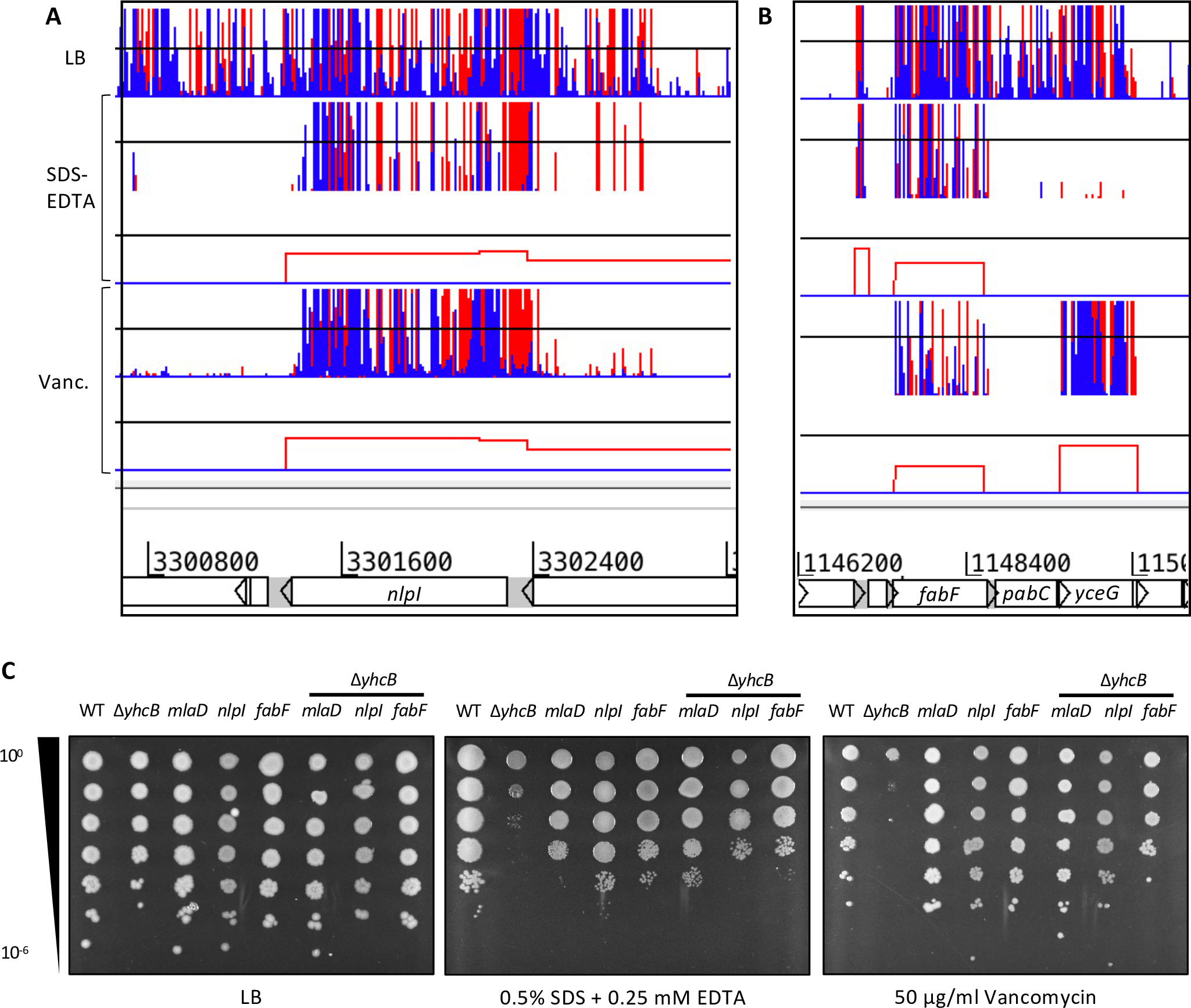

Supplement: S8 Fig — (A and B) Transposon insertion data for suppressor screens. Red and blue vertical lines indicate the transposon insertion position and correspond with the transposon orientation at the point of insertion. The height of the bar corresponds with mapped sequencing read frequency. The top track represents data for the library plated on LB. The tracks underneath represent the ΔyhcB transposon library plated on LB supplemented with SDS and EDTA, or vancomycin, at lethal doses to the BW25113ΔyhcB parent strain. The red boxes underneath each suppressor dataset represent significant differential abundance of insertions in the condition sample compared to the control, identified by AlbaTraDIS. (C) Overnight cultures of the wild-type and ΔyhcB strain grown alongside isogenic single and double gene-deletion mutants constructed by P1-transduction using the Keio library strains as donors. Cells were grown in LB at 37°C, normalised to an OD600 of 1.00 and 10-fold serially diluted before inoculating LB agar plates supplemented with SDS and EDTA or vancomycin. (TIF) [file pgen.1009586.s021.tif]

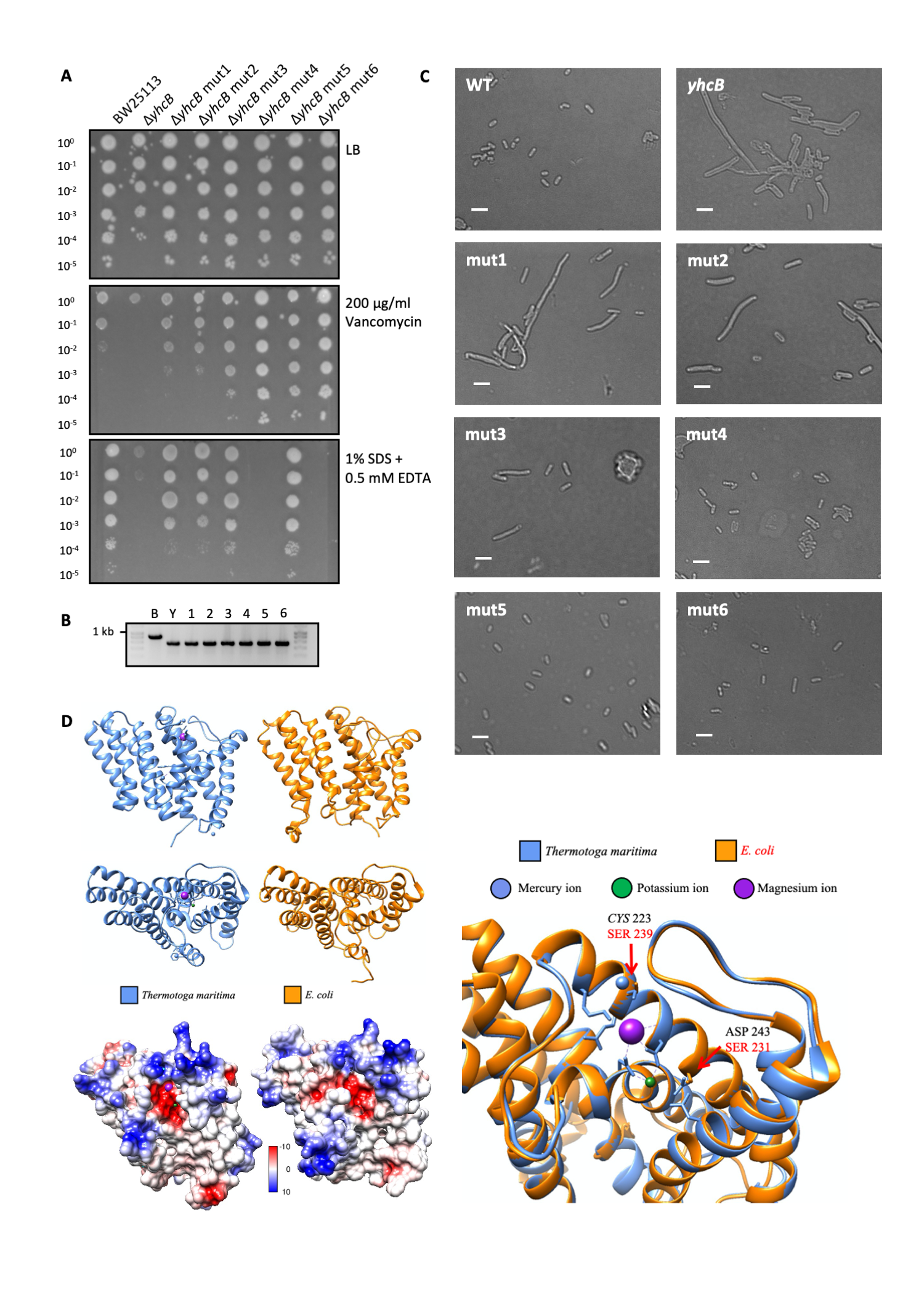

Supplement: S9 Fig — (A) Validation of natural suppressor mutants. 10-fold serial dilutions of E. coli K-12 BW25113, BW25113ΔyhcB and six BW25113ΔyhcB revertant suppressor mutants, grown on LB, LB supplemented with 200 μg/ml vancomycin, or 1% SDS + 0.5 mM EDTA. (B) PCR amplification of the yhcB locus to confirm yhcB-deletion in these strains. B = BW25113; Y = BW25113ΔyhcB; 1–6 = BW25113ΔyhcB suppressor mutants 1–6. (C) DIC images of BW25113, BW25113ΔyhcB and six BW25113ΔyhcB revertant suppressor mutants grown overnight in LB. Scale bar of 5 μm. The precise genotype for each mutant is listed in S7 Table: mut 1 has a single nucleotide polymorphism in lpxC; mut 2 has a genomic inversion resulting in an mla null phenotype; mut 3 and 5 have single nucleotide polymorphisms in cdsA; mut 4 and 6 have mutations consistent with the previously described mla*. (D) Solved structure of Thermatoga maritima CdsA (blue) and the predicted structure of E. coli CdsA (orange), with the surface electrostatic potential of each shown below. The deep red and deep blue colours indicate electronegative and electropositive regions at −10 and 10 kT e−1, respectively. An overlay of the cation-binding pocket of CdsA from T. maritima and E. coli is shown in the bottom right panel. The equivalent mutated residues are annotated: shown in black, above for T. maritima and red, below for E. coli. Note the S223C residue mutated in T. maritima to achieve a resolved structure. (TIF) [file pgen.1009586.s022.tif]

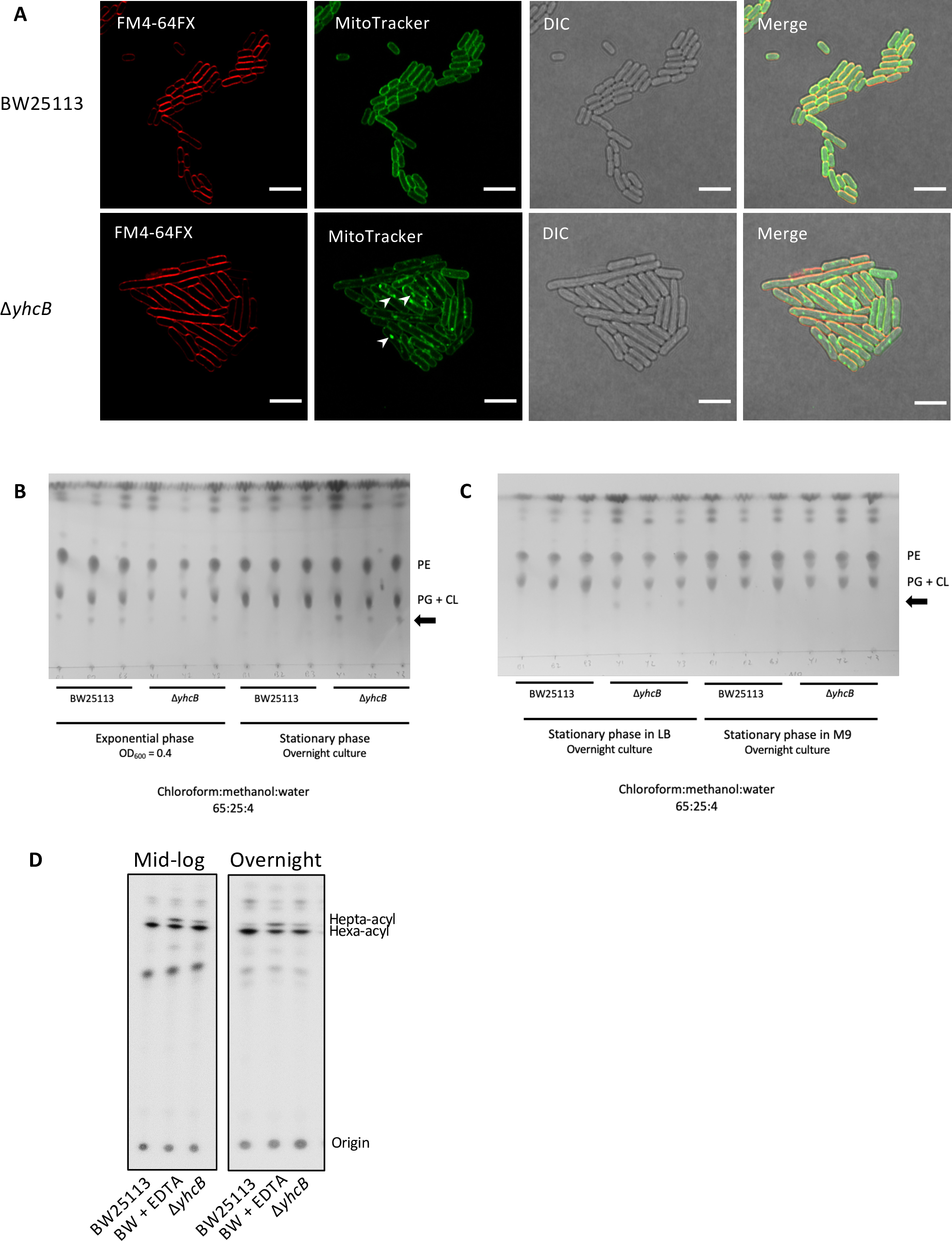

Supplement: S10 Fig — (A) E. coli K-12 BW25113 and BW25112ΔyhcB grown in LB, labelled with FM4-64FX and MitoTracker Green FM. Images were taken at 3 h and are representative of n = 3 experiments. Lipid accumulation is indicated by the white arrows. Scale bar of 5 μm. (B-C) Thin layer chromatography (TLC) separation of phospholipid species in chloroform:methanol:water (65:25:4) solvent system, and stained with PMA. (B) Samples grown in LB and collected at two stages of growth. (C) Samples grown overnight in LB or M9-glucose. (D) TLC/autoradiographic analysis of [32P]-labeled lipid A extracted from mid-logarithmic growth and overnight growth cultures of BW25113 and ΔyhcB strains grown in LB. As a positive control for lipid A palmitoylation, BW25113 cells were treated with 25 mM EDTA for 10 min prior to extraction. Abbreviations: Phosphatidylethanolamine (PE); Phosphatidylglycerol (PG); Cardiolipin (CL). (TIF) [file pgen.1009586.s023.tif]

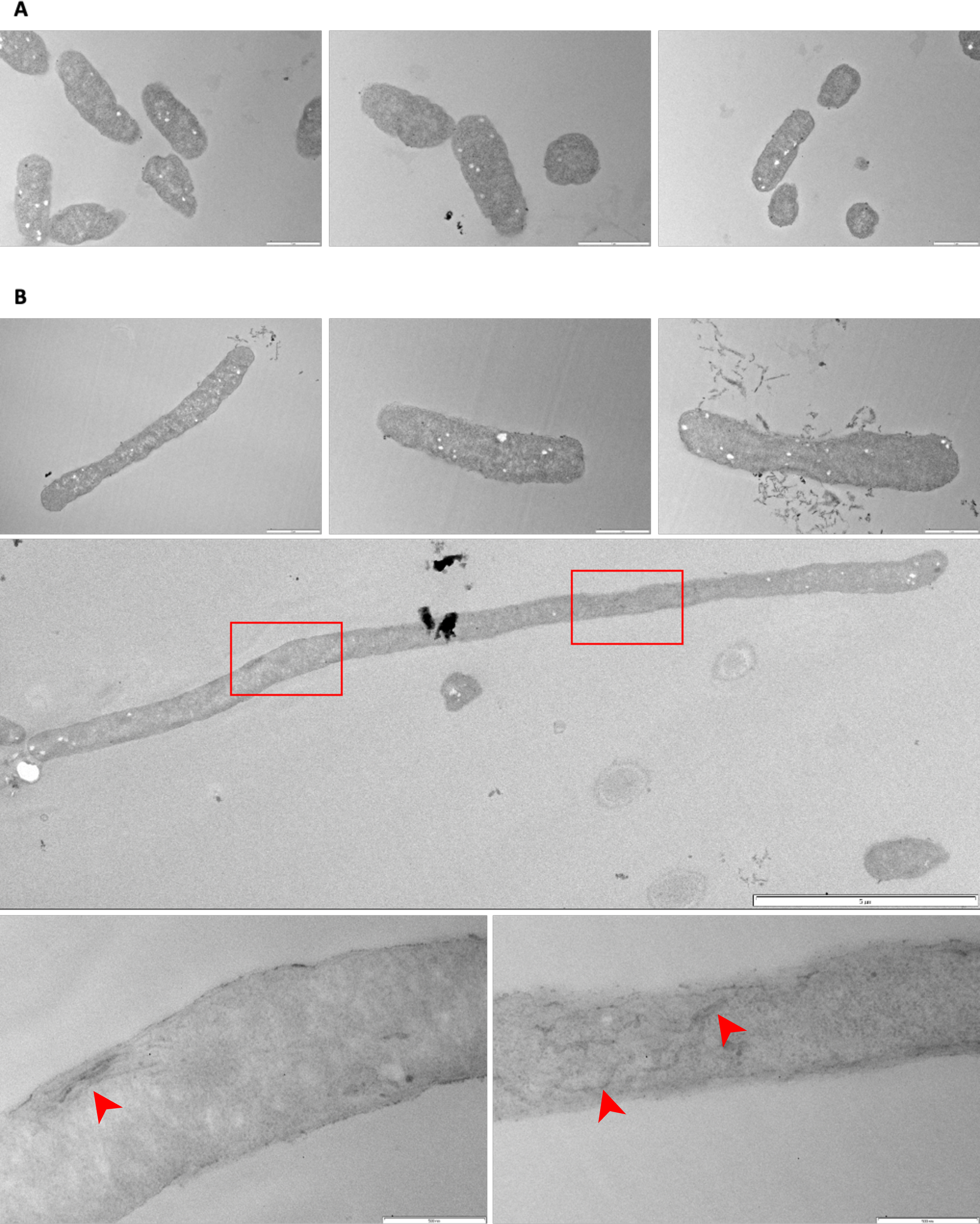

Supplement: S11 Fig — Transmission electron micrographs of (A) BW25113 and (B) the ΔyhcB mutant cells (standard fixation and processing). Scale bar = 1 μm in the top six images. A large ΔyhcB mutant cell is shown with a scale bar of 5 μm and two regions of excess or ruffled membrane structures are highlighted by red boxes (annotated in Fig 10A); enlarged images of these sections are shown underneath with a scale bar of 500 nm each. (TIF) [file pgen.1009586.s024.tif]

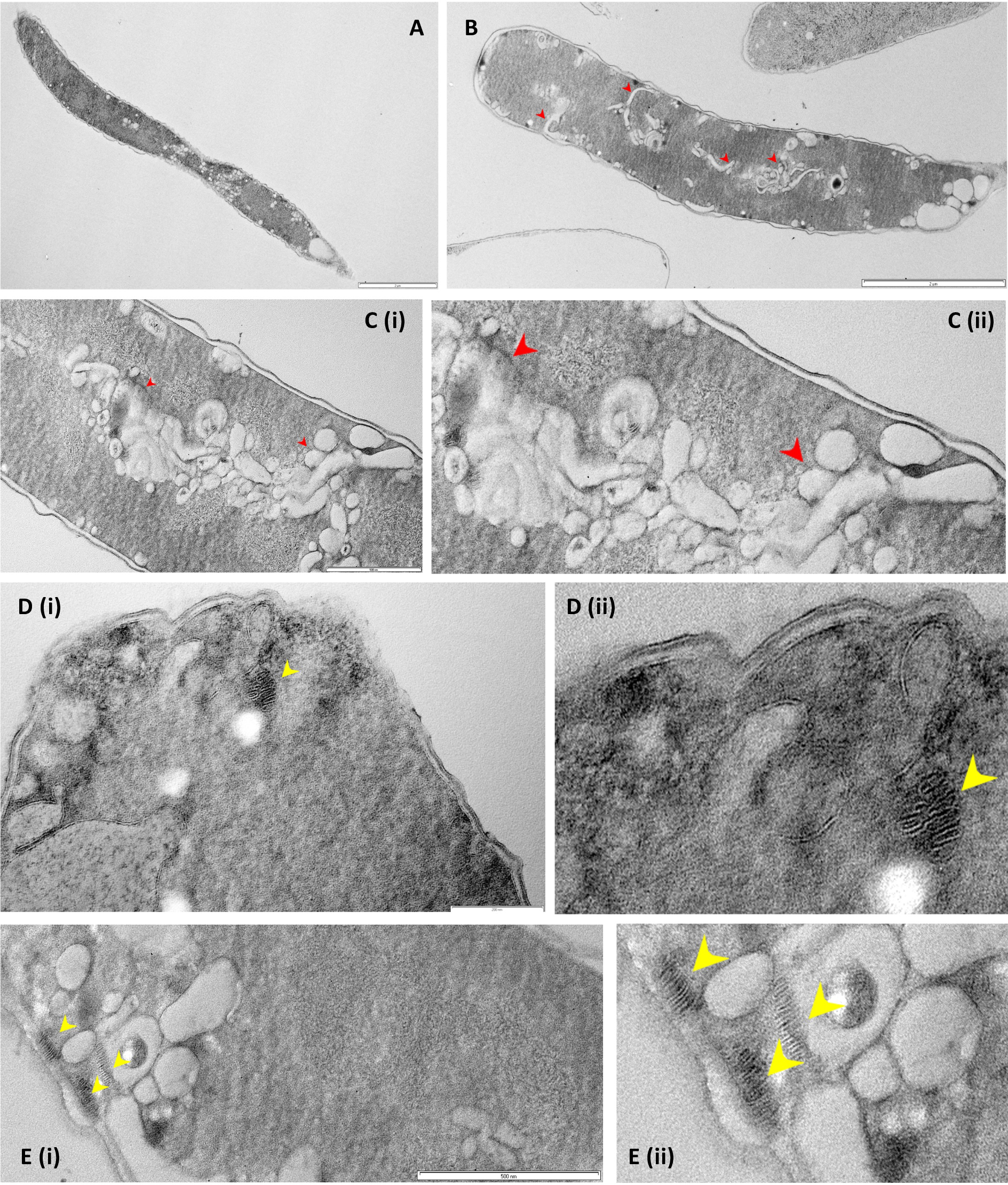

Supplement: S12 Fig — Electron micrographs of fast frozen/freeze-substituted yhcB mutant cells. Internal membranes indicated by red arrowheads; stacked membrane arrays indicated by yellow arrowheads. Bars are: (A) 2 μm; (B) 2 μm; (C) 500 nm; (D) 200 nm; (E) 500 nm. Note that panels C-E (ii) are higher magnification views from C-E (i), respectively. (TIF) [file pgen.1009586.s025.tif]

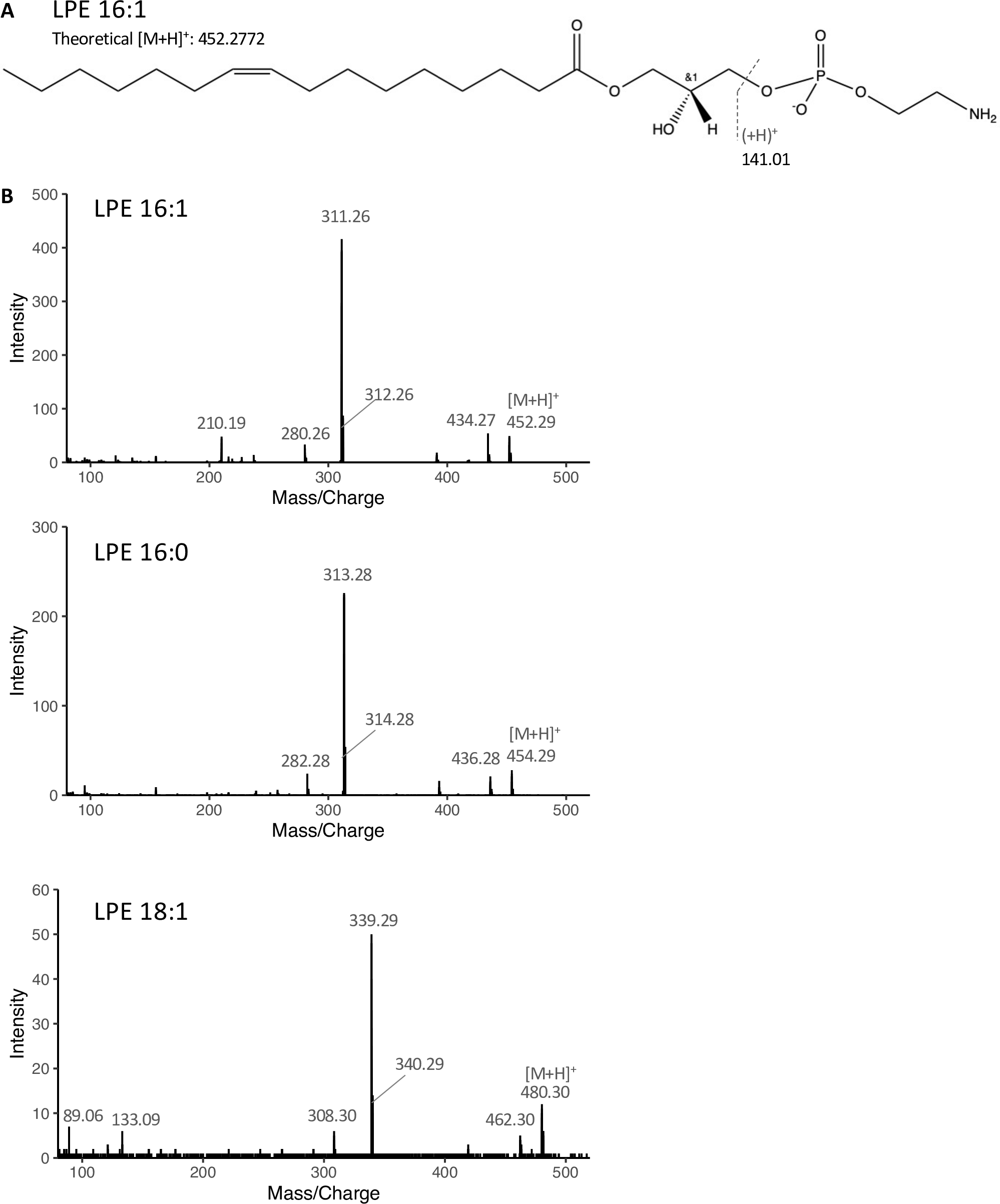

Supplement: S13 Fig — (A) The chemical structure of lyso-phosphatidylethanolamine (LPE) 16:1. (B) Product ion ms/ms spectra of the three most abundant LPE species detected by mass spectrometry. The spectra show characteristic loss of 141 Da from the molecular ion [M+H]+, corresponding to the loss of the PE lipid head group. (TIF) [file pgen.1009586.s026.tif]

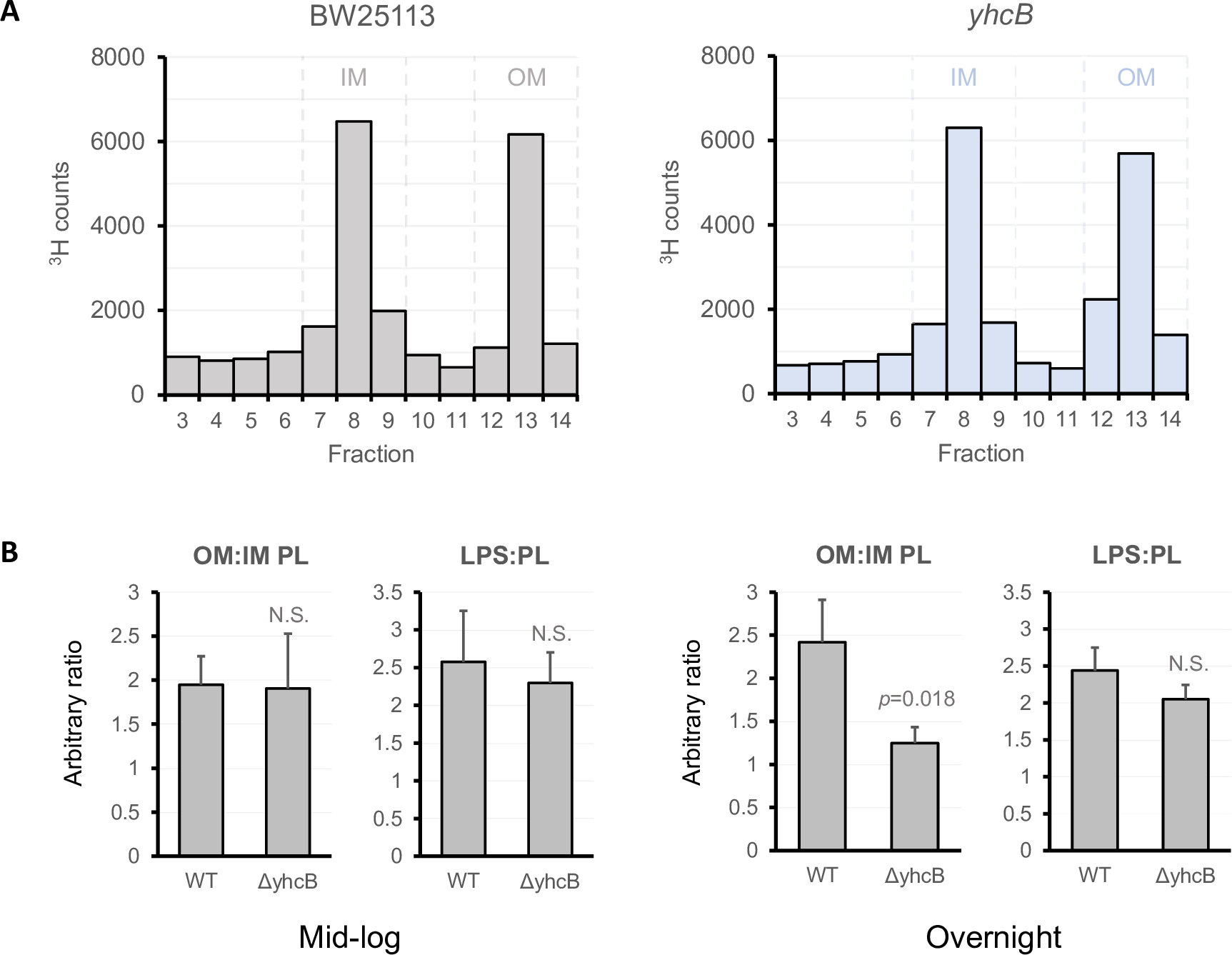

Supplement: S14 Fig — (A) A representative [3H]-distribution profile of cell lysate fractions separated by centrifugation across a sucrose gradient, taken from cell cultures grown overnight in LB. (B) Ratio of [32P]-phosphate labeled phospholipids (PL) between the outer membrane (OM) and inner membrane (IM), and between OM LPS and PLs, for both exponential and stationary growth phase cell cultures, in triplicate. Error bars represent standard deviations. Student’s t tests: N.S., not significant, as compared to wild type (WT). (TIF) [file pgen.1009586.s027.tif]

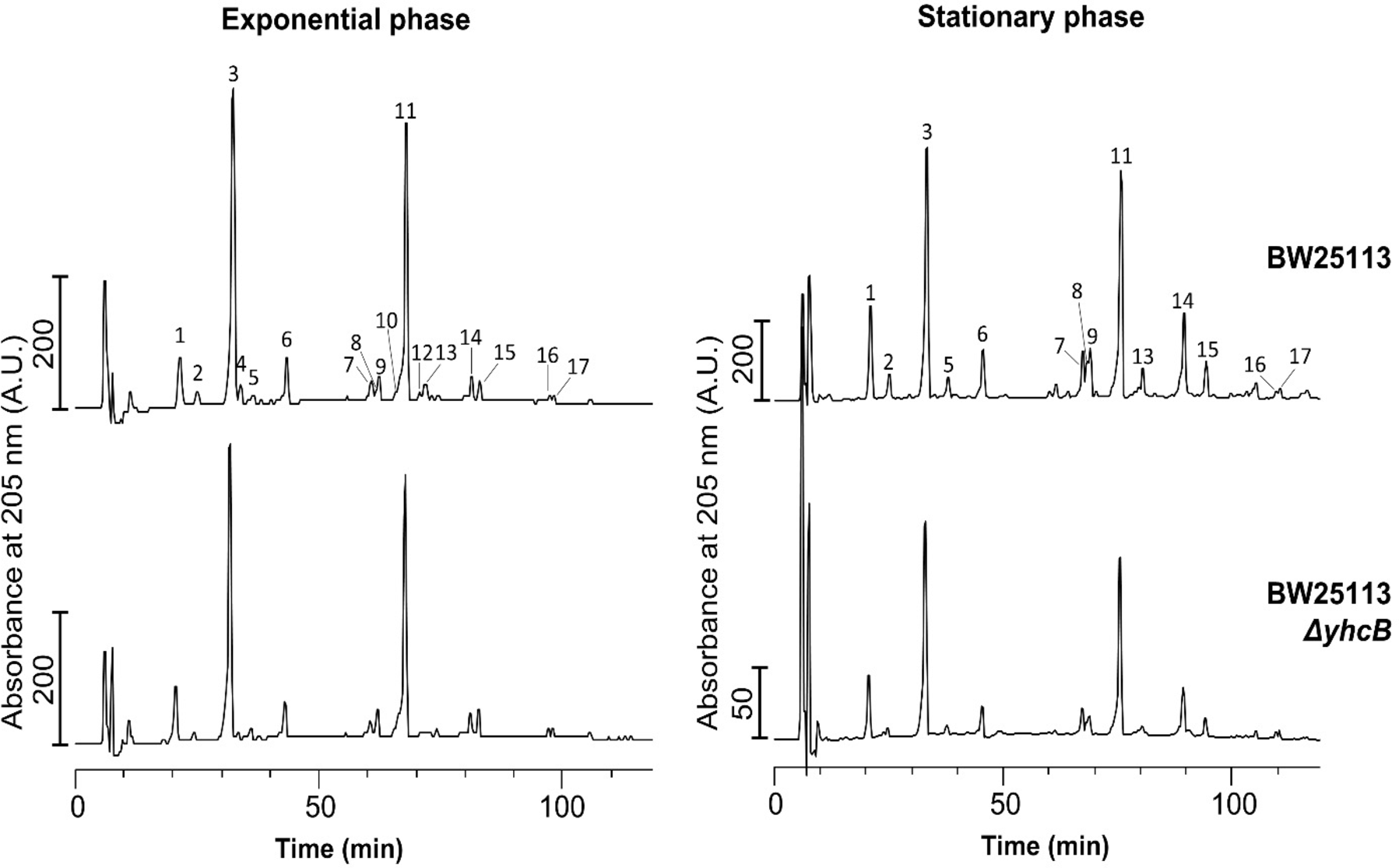

Supplement: S15 Fig — Representative HPLC chromatograms showing the muropeptide composition of BW25113 and BW25113ΔyhcB at exponential and stationary phase. Purified peptidoglycan was digested with cellosyl and the resulting muropeptides were reduced with sodium borohydride and separated by HPLC. The major muropeptides (No. 1–17) are quantified in S9 Table. (TIF) [file pgen.1009586.s028.tif]

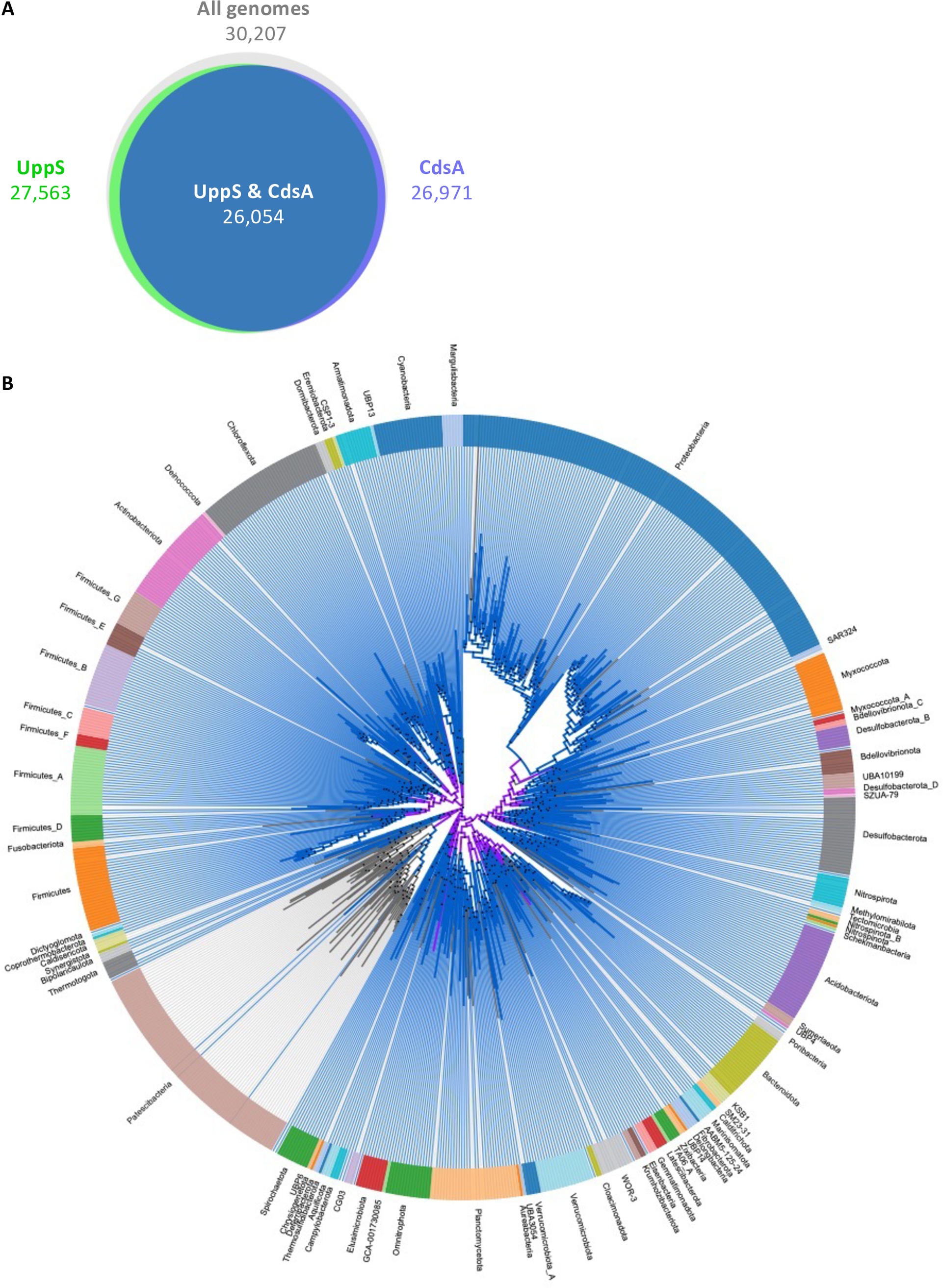

Supplement: S16 Fig — The number, and overlap, of bacterial genomes containing a UppS (K00806) or CdsA (K00981) homolog obtained from AnnoTree (v1.2.0) using the KEGG identifiers and the following search criteria: % identity: 30; E value: 0.00001; % subject alignment: 70; % query alignment: 70. (B) Tree representation of the phylogeny of bacterial genomes with species containing both UppS and CdsA highlighted in blue. (TIF) [file pgen.1009586.s029.tif]

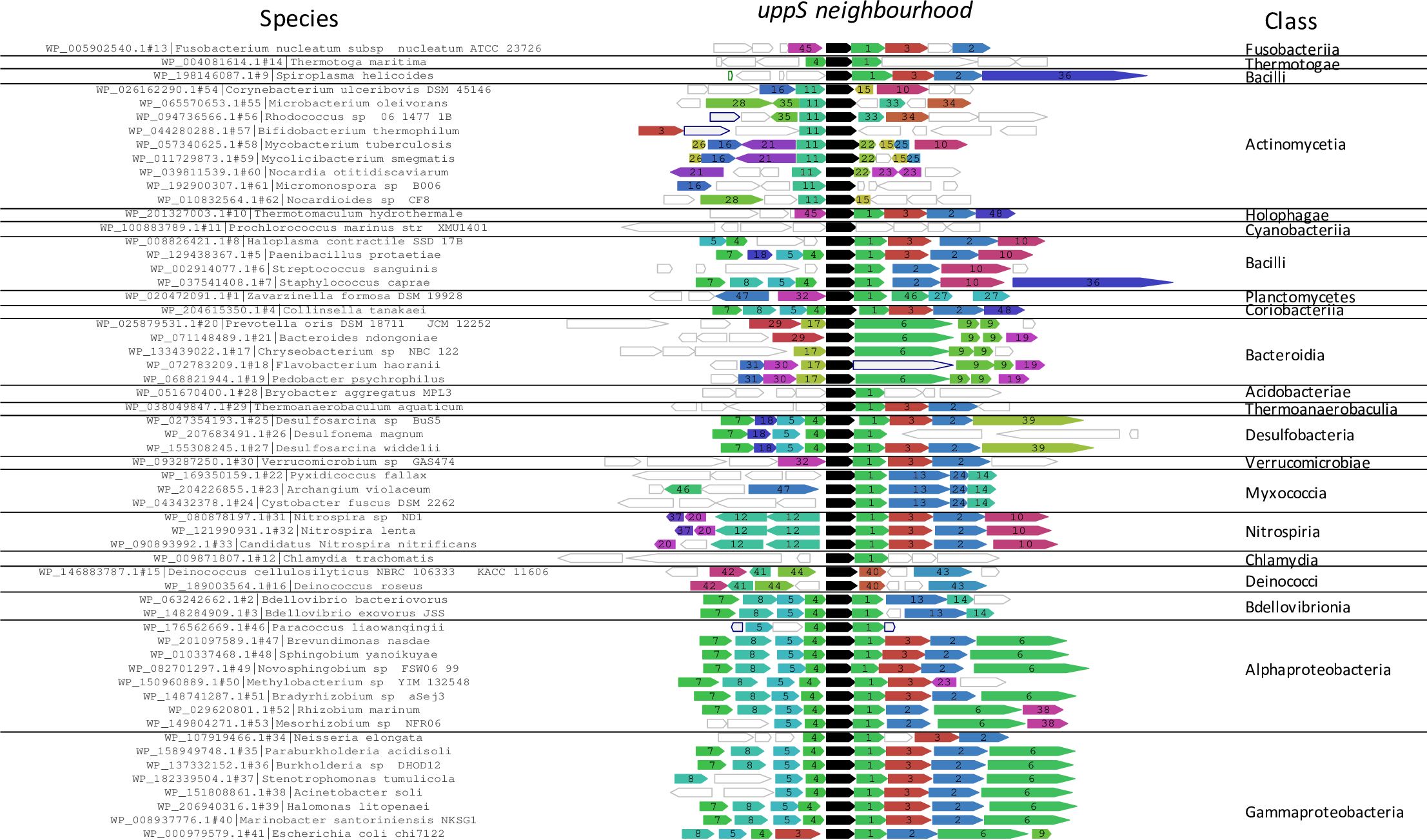

Supplement: S17 Fig — The conservation of the uppS gene neighbourhood represented by 62 diverse bacterial species. uppS is depicted in black, and cdsA in green (1). The remaining gene identifiers can be found in S10 Table. Data calculated using FlaGs [109]. (TIF) [file pgen.1009586.s030.tif]

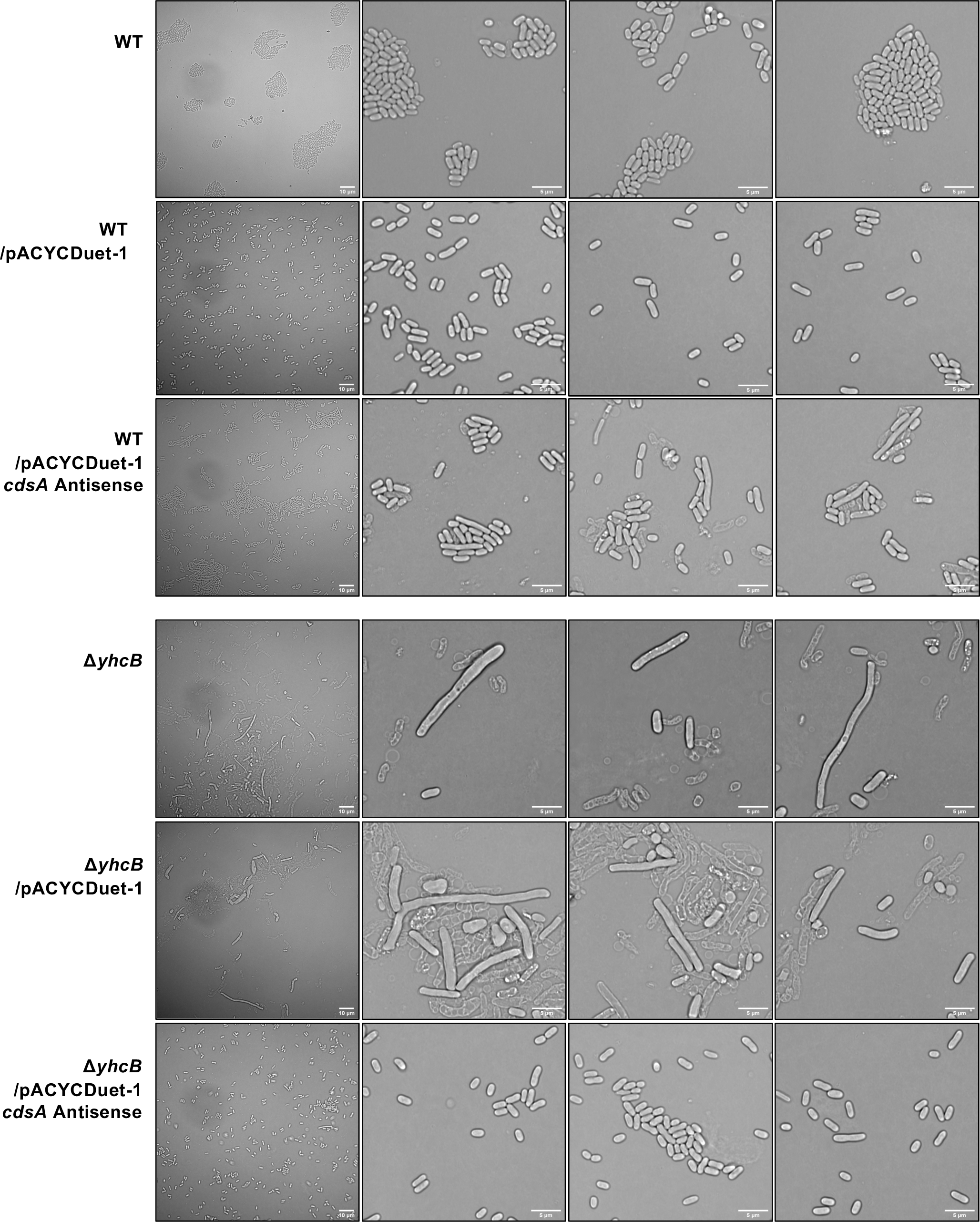

Supplement: S18 Fig — DIC images collected on the Inverted LSM 880 Fast Airyscan (63x/1.40 OIL) for stationary phase cultures grown overnight in LB at 37°C without induction. Leaky expression of the antisense cdsA transcript from the T7 promoter was sufficient to affect changes in morphology. Scale bar = 10 μm in column A, scale bar = 5 μm in columns B-D. (TIF) [file pgen.1009586.s031.tif]
