# Supplementary material for: Loss of YhcB results in dysregulation of coordinated peptidoglycan, LPS and phospholipid synthesis during Escherichia coli cell growth
Source: PLoS Genet. 2021 Dec 23;17(12):e1009586. doi: 10.1371/journal.pgen.1009586 (PMC8741058; doi:10.1371/journal.pgen.1009586)
Supplement: Attachment — Submitted filename: YhcB response to referees comments.docx [file pgen.1009586.s032.docx]

We thank the referees for their thoughtful and insightful comments. We have addressed all their concerns below, and have incorporated some additional work into the manuscript to bolster our observations.

Referee 1.

*1) In my opinion, the manuscript should focus on the novel data presented here and the role of YhcB on lipid synthesis. Figures 2-4 could be supplementary or deleted given that they have been reported by others.*

We thank the referee for these recommendations. However, it is important to note that the work presented by Mehla et al was published while this manuscript was under review. Nevertheless, the data presented in original Figure 4, with the exception of one construct (YhcBΔTM), has never been shown before. Figures 2 and 3 have been combined to condense this section, the novelty and significance of this data has been addressed below and in response to comment 8 from the reviewer. For example, Sung *et al.* demonstrated that loss of *yhcB* affects cell length, but this data was limited to just growth in LB medium we revealed a growth medium effect. At the time of submission, this manuscript was the first body of work connecting growth temperature and media with phenotype. Work presented by Mehla *et al.,* demonstrated growth in rich media at 37˚C or minimal medium at 28˚C; this manuscript clarifies that both a reduced temperature and minimal media are independently restorative. Our manuscript also goes a step further by demonstrating that growth in minimal medium is not restorative of all phenotypes. Lastly, this manuscript demonstrates the decrease in viability observed by Sung et al occurs upon transition to stationary phase. These new findings are presented in the now combined Figure 2.

*2) Genome-wide screens like the ones reported here which search for mutations that display either a positive or a negative synthetic effect in an yhcB mutant are powerful, but they should be complemented with more detailed analyses of the genetic interactions revealed by the screens. This manuscript fails to do this. As a result, it is difficult to understand the extent of the negative and positive interactions reported in this work, despite the strong conclusions stated by the authors. For example:*

*a) Are the combinations deemed synthetic lethal in Figs. 6 and 7 truly lethal (as the authors state) or do the double mutants grow significantly worse than the single mutants?*

We thank the reviewer for their feedback. We wish to note that the selection method used allows for recovery of slow growing mutants, without competition. However, recovery of slow-growing mutants was capped at a timespan of 18 h. Transposon Insertion Sequencing (TIS) methods are exceptionally sensitive and even small colonies can be identified by sequencing. It is possible that colonies that require longer than 18 hours to develop may have been missed by our selection criteria. However, this becomes a more philosophical debate centred around the definition of a severe genetic interaction, and whether double mutations that can be rescued by compensatory mutations can truly be considered ‘synthetically lethal’. One point worth noting is that TraDIS data are quantifiable, each gene in the *yhcB* library has a logFC score and associated Q-value, giving a measure of the strength of the positive/negative interaction.

Secondly, given the density of this library (1 insertion/ 7 bp), while there might be stochastic loss of individual mutants, a complete absence of mutants (of any given gene) from the output library gene does indicate a synthetic lethal relationship. An internal validation for this library was the already published synthetically lethal relationship demonstrated between YhcB and RodZ (PMID: 27335665).

Lastly, we acknowledge that no method that involves screening thousands of mutants has a perfectly binary output of lethal/essential and viable/non-essential growth, as has been noted in many high-throughput essentiality and TIS screens. For this reason, we applied doubly stringent criteria for the identification of ‘synthetically lethal’ genes. Specifically, we required both a statistically significant underrepresentation of mutants (i.e. an insertion frequency that would result in a gene being classified as ‘essential’) in addition to a >2-fold decrease in insertion frequency compared to the control dataset, thereby differentiating ‘sick’ from ‘lethal’ mutations. In support of our TIS data, we have included validation experiments for a representative gene from the key pathways examined in this manuscript.

*b) Is an ftsH null allele truly not lethal in a yhcB mutant unlike in the wild type? The incidence of transposons in ftsH is still quite low in the yhcB mutant. Given that there are known suppressors of the essentiality of ftsH, the low incidence of transposition might indicate that the ftsH yhcB mutant has acquired additional mutations that allow its survival.*

To address this point, we deleted *ftsH* in a Δ*yhcB* mutant. See Figure S6.

*c) How strong is the suppression reported by the loss of Mla? Are the phenotypes (TEM and lipid changes) reported in Fig 10 for the yhcB mutant corrected by mla mutations?*

We thank the referee for highlighting this. Fig. S9A-C already demonstrates that separation of the mla operon from its promoter restores growth of a *yhcB* mutant in the presence of vancomycin, and SDS-EDTA, but not cell size. To further support our TIS data and the natural revertant suppressor data, we constructed a double Δ*yhcB*Δ*mlaD*::*aph* mutant and found the double deletion restored SDS-EDTA tolerance to WT levels (Fig. S8C). Furthermore, the double mutant was more resistant to SDS-EDTA than either single mutation. Although it would be interesting to examine the phospholipid content of each of the suppressor mutants, it is beyond the scope of this manuscript.

*There is a lot of data included in the manuscript because of the nature of the screens, but the depth of knowledge gained from this study is limited because the authors do not analyze mutants in depth. The exception is the yhcB single mutant, but a lot of that data has already been published (as the authors cite, although do not always make clear).*

The referee has been kind to point this out but as we highlighted the work presented by Mehla et al was published while this manuscript was under review. We had good fortune to share our prepublication data with those authors in January and those discussions enhanced the research of both groups. In both cases the focus of the manuscripts is on *yhcB* and we are sure a myriad of future PhD students and postdoctoral fellows will be able to devote time to analysing the significance of other genetic interactions in the future.

*3) The synthetic genetic interactions suggest that yhcB mutants have an imbalance in the synthesis of total levels of phospholipids, LPS, and undecaprenyl phosphate, but the authors do not address if this is the case. Are the total levels of LPS and phospholipids changed in the yhcB mutant? If affected, they could also test to see if suppressors fix the imbalance.*

This is a very valid point and we thank the referee for raising it. We quantified the relative abundance of phospholipid:LPS at the OM, in addition to the relative amounts of phospholipid between the IM and OM and have addressed this point in the text (Fig. S14). None of the natural suppressor mutations (mut1-6) abolished the accumulating LPE spot when evaluated by TLC. The latter point however was not included in the manuscript as it added little to the narrative to a manuscript that is already long. Quantification of Und-P would be the logical next progression and is of interest for the future direction of this work, however our genetic work support this observation.

*4) Are suppressors of sensitivity in an yhcB mutant specific to the loss of YhcB or are they mutations that always increase resistance? The authors use the suppressor data to make their case for a role of YhcB in coordinating lipid synthesis. However, given that the suppressor screen was for increased resistance, their argument would be strengthened if suppressors were shown to be specific to the loss of YhcB. For example, it was previously reported (but not noted in the manuscript) that cdsA mutations confer a general increase in vancomycin resistance, even in a wild-type cell (PMID: 24957626)*.

Here we agree with the reviewer that a suppressor screen has the potential to be skewed by selection for merely ‘more resistant’ mutants. However, to combat this in our experimental design we screened at concentrations lethal to the parent Δ*yhcB* strain. Setting a high (life or death) threshold for suppression, identifying only dominant mutations that were able to bypass lethality, was one measure we implemented to minimise false positives. Secondly, we focused our analysis on those mutations able to restore 2 or more phenotypes, on the grounds that a mutation able to restore viability under two chemically diverse conditions is increasingly likely to be functionally related. As was noted in the manuscript with the identification of *mla* mutations, which notoriously confer increased vancomycin resistance but are highly sensitive to SDS-EDETA.

In support of our TIS data, we validated our findings by construction of targeted single and double gene-deletion mutants for the most prominent suppressor mutations. Screening of these mutants corroborated the TIS data.

*5) Discussion lines 757-591: There is no data demonstrating that they have decoupled these phenotypes because the authors only measured growth at one concentration for each treatment. Sensitivity could be decreased somewhat but not enough to make a difference in growth in the specific concentration used by the authors. If they are going to make this claim, the authors should assess sensitivity using more quantitative methods (like minimal inhibitory concentrations). This also applies to the data from Fig 4 and the conclusions regarding the PRDY domain of YhcB only being required for resistance to some compounds. Otherwise, what’s the explanation for the difference?*

We thank the referee for making this important point, and agree that the sheer volume of data in this manuscript sometimes makes it difficult to integrate all of the data logically. Here we highlight that each stress-screen has a different collection of genes, albeit with some overlap, that are able to suppress the *yhcB* phenotype. Therefore, this suggests that the phenotypes can be decoupled at the genetic level. Second, we have shown a decoupling of the cell size defect and resistance to chemical stresses (Fig. S9). We are unsure how determining an MIC would add any further value to our observations given that in each case the concentration of compound was one that ordinarily kills the yhcB-deletion strain but was non-toxic to the BW25113 strain; such observations would only capture data to differentiate the strength of suppression. The inhibitory concentrations of the compounds used for the later stress screening experiments had already been established for this strain in Fig. 2A. We are unsure of the meaning in the referees final point.

*Minor comments:*

*1) Line 28: Change “kingdoms” to “domains”.*

Done

*2) Line 31: change “most well” to “best”.*

Done

*3) Line 34: Change "whole genome phenotypic" to "whole-genome phenotypic".*

Done

*4) Line 38: The authors state that they report “the complete” genetic interaction network of yhcB. It might be complete for transposon insertion mutants, but that is all. There might be other members of the network that could not be found because of the use of transposon mutagenesis. Remove “the complete”.*

changed to “we report the comprehensive genetic interaction network of yhcB”

*5) Line 42: I suggest changing "sits” to " functions".*

Done

*6) Lines 51-52: Doesn't the cell envelope also include the periplasm?*

In Gram-negative bacteria, the tripartite cell envelope is composed of the cytoplasmic membrane (CM), the peptidoglycan (PG) layer within the periplasmic space and an outer membrane (OM)

*7) Lines 62-77: The references used to cite the machineries that are involved in cell envelope biogenesis, elongation, and division need to be revised. Several of the cited reviews are from 5-10 years ago, so newer ones should be considered. In addition, the referencing of the primary literature is incomplete. The authors cited papers reporting the discovery of some but not all the components of the various machines.*

We have included additional references and more recent reviews, where available.

*8) Lines 92-93: The authors state that they demonstrate that loss of YhcB affects cell width and length but do not reference that this was shown previously by others. The images here are similar to those shown by Sung et al. However, Mehla et al reported increased length but reduced diameters. Can the authors comment on this difference?*

We thank the reviewer for the opportunity to clarify this point, and also wish to highlight again that the Mehla et al manuscript was not published at the time of submission of this manuscript. Sung *et al.* (2020) demonstrate that a MG1655ΔyhcB mutant grown in LB has a cell length defect. However, they do not quantify the mutant cell width. Moreover, Sung et al. report a subset of the population display a cell-branching morphology, when grown in LB. We have also observed this phenotype. The data presented by Mehla *et al.,* (2021) specifically the finding of ‘narrower’ cells, is at odds with the data presented by Sung *et al.* demonstrating variable and non-uniform cell widths (branching) in Fig. 6A. Moreover, the DIC images presented by Mehla *et al.* in Fig. 6A suggest the *yhcB* mutant is indeed wider when compared to their control, but this is in direct contradiction of their phase contrast microscopy images in Fig. S2A. The methodology presented by Mehla *et al*. states “ImageJ software was used for measuring cell dimensions/length”, it would be beneficial to understand how their width measurements were calculated (line measurement, vs. intensity etc) in order to further understand the discrepancies in the literature. However, Sung *et al.* constructed a Δ*yhcB* mutant via lambda recombination, whereas Mehla *et al.* used the Keio library mutants. We have observed that the Keio library *yhcB* mutants do not phenocopy each other, and through whole genome sequencing have identified that both Keio *yhcB* mutants contain different sets of second site suppressor mutations. Given the sites of these mutations, they likely alter growth rate, and may affect cellular morphology. Finally, we are confident in our data and its rigour, however we cannot at this time give a clear explanation for the differences between our work and that of another lab with whom we were not integrally involved.

*9) Line 95: delete “all”. How do you know that you found all genes that suppress the envelope defects caused by the loss of yhcB? Possibly transposon mutant alleles, but not necessarily all genes.*

We are unsure of the point the referee is making given that the frequency of mutation is 1/7 bp. However, we have rewritten the sentence for clarity “Subsequent screening of this library against chemical stresses revealed all mutations of this library that suppress the envelope defects associated with the loss of yhcB”

*10) Line 97: I suggest changing "is stationed" to "functions".*

Changed to “positioned”

*11) Fig. 2A: the strains label for the mutant is missing and the wild-type strain's label is misplaced.*

Fixed

*12) Lines 154-156: It appears from the images on Fig S2A that over-production of YhcB is somewhat toxic in wild-type and mutant strains. I suggest removing any description about length and instead comment on the detrimental effect on growth. The effect in length is not very convincing based on the data shown*.

We have deleted “did not restore cell size completely” from the text. We have also addressed this point in detail in response to Reviewer 2, point 1d. But wish to respond to two points here. 1) Given the Δ*yhcB*/p*yhcB* strain is more fit, despite the transparent morphology, than the Δ*yhcB/p-*emptyvector strain when exposed to SDS-EDTA, we do not consider a transparent morphology as sufficient evidence of toxicity. 2) The scale bars in the micrographs are of uniform length (5 µm) as noted in the figure legend, we therefore disagree that the effect on length is not convincing given the substantial change in cell size of the Δ*yhcB* mutant when p*yhcB* is expressed.

*13) Lines 186-188: Do they lose viability or is it that because they filament they are expected to yield a lower number of colonies?*

The decrease in CFUs (rather than plateau) suggests cell death. This hypothesis is supported by the propidium iodide live/dead staining and microscopy data reported by Sung et al. (2020) in Fig. S4, showing a larger proportion of *yhcB* cells stained with PI under ‘normal growth conditions’.

*14) Line 218: remove “all”.*

Done

*15) Line 301: What is meant by LPS stability?*

We have altered the text to read:

Together these data suggest that OM rigidity and structural integrity, whether mediated by Lipid-A hexa-acylation or LPS crosslinking interactions, is important for viability of a Δ*yhcB* strain.

*16) Fig. 9 suggests that there is a preferred directionality in transposon insertion based on the color of the transposon (red is predominant, whatever direction that is since information is not provided; likely internal promoter drives expression of the locus). Is there a preference in directionality? If there is, the authors should comment on it. Later, there is data on uppS and cdsA that should have been mentioned here as well.*

This is well observed by the reviewer. We have added details of the transposon directionality in the legend. There is a directionality preference under the SDS-EDTA selection conditions, but no preference (both orientations are favoured) under the vancomycin selection conditions. For simplicity we have added ‘It was unclear from the insertion pattern the effect that disruption at this locus would have’ and followed this up with validation experiments later in the manuscript.

*17) Lines 374-392: The nature of the mla* mutation should be explained in more detail to readers. Also, May et al. was not the original paper reporting the mla* allele, the Sutterlin et al 2016 PNAS paper was.*

We thank the referee and altered the text as follows:

The *mlaA** mutation is well characterised and is toxic in a WT background as it results in a flux of phospholipids into the OM outer leaflet, which triggers PldA-mediated phospholipid degradation and a fatty acid-mediated signalling cascade that results in increased LpxC stability and increased LPS production (Sutterlin et al. 2016; May and Silhavy 2018)

*18) Line 381: what does a variation of mlaA* mutation mean? Be specific.*

This information was specifically provided in former supplementary table 8 (current Table S7), which reports the precise mutation in each mutant.

*19) MlaA* works in the opposite way as wild-type MlaA. How do the authors explain that both mlaA* and mlaA nulls suppress?*

Phospholipid synthesis takes place at the IM. It is our hypothesis that loss of *yhcB* is resulting in an increase in phospholipid synthesis, which is supported by our phospholipid quantification data, our TEM images that show excessive phospholipid accumulation in the *yhcB* mutant occurring internally, and the RT-pCR indicating overexpression of cdsA. It is our hypothesis that the *mlaA** mutation can partially suppress this defect as it increases the flow of phospholipids away from the IM. Accumulating PLs at the OM outer leaflet will be degraded, as well as triggering an increase in LpxC stability and concomitant increase in LPS synthesis. We don’t know the precise mechanism for how loss of the *mla* pathway is restorative to a *yhcB* mutant. One explanation is that loss of the *mla* pathway may limit the retrograde transport of PLs to the already overburdened IM. Another hypothesis is that disruption to the *mla* pathway may enable shedding of the excess phospholipid material via vesiculation at the OM. However, this warrants further study.

*20) Line 438: Fig. S10 should be S12.*

Done

*21) Line 486: Fig. S10D should be S12D. In addition, this figure needs to include a control showing what happens when the wbbL plasmid is introduced into the wild-type strain to make sure that their conclusion is warranted. Are the colonies growing suppressors ?*

This section has been deleted without follow up.

*22) Lines 502-504: I suggest deleting the sentence “With … resolution”.*

We have retained this statement as we believe it to be true.

*23) Lines 506-508: Rephrase this sentence as it is confusing. The data support that the defect is most severe in LB than in minimal, but not during rapid growth in rich media. As the authors state in their next sentence, defects are most severe during stationary phase in rich media.*

The sentence has been changed to read “The defect introduced by deletion of yhcB is most severe following rapid growth in rich media…”

*24) Lines 513-517: I am confused by the reference to Harris and Theriot since the yhcB mutant has both increased diameter and length, so the volume is greatly affected and not maintained.*

These lines have been deleted.

*25) Fig. 11 and legend: References showing interactions should be cited in legend. What is the difference between dotted and solid arrows?*

Arrow descriptions have been added to the legend. All interaction references are already discussed and cited in the text. Citations for the interaction partners of YhcB have been added to the figure legend.

*26) Line 533-535: The images presented by the authors showing the effect in size (Fig, 2) do not resemble those shown in Vadia et al 2017. The yhcB mutants appear to have a septation defect (as shown by others), while those in Vadia did not.*

Please refer to the cells shown in Figure S5 of Vadia et al 2017 when induction of FadR is greatest.

*27) Line 557: “Given the location of YhcB”… What do the authors mean by this? The reported co-localization with the divisome reported by Mehla et al?*

We have altered the sentence for additional clarity. Given the location of YhcB within the IM, its interaction with the elongasome, and the phenotypes of a deletion mutant

*28) Line 593: remove the reference to “all of the cell envelope biogenesis pathways”. There is no evidence that YhcB affects all pathways.*

‘all’ has been deleted.

*29) Methods: More detailed explanation of plasmid constructions should be given.*

Reviewer #2:

Main comments:

*a) Fig 2A. Please label the mutant sample; I assume this is the right dilution column in each plate panel.*

Done

*b) There is a clear difference in LB versus M9-Glc in Fig 2A to the extent that SDS/EDTA arguably has little effect in cells grown in M9 Glc. The way the data is described in lines 139-147 fits the LB phenotype but not M9-Glc. Some (but not all) of this is clarified later but it was frustrating to try and sort it out while working through the results sequentially*

This section has been edited slightly for clarity, in addition to adding (i) and (ii) labels to the figure to make it clear when each part of the plate selection is being referred to.

*c) The text implies the complementation data is in Fig 2A but it isn’t. Fig. S2A needs to be referenced in the sentence that begins on line 144 “Ectopic expression….”.*

Done

*d) Fig S2A (right) shows an effect of transforming the WT with pBAD-yhcB that goes away with Ara induction. Please explain this observation.*

We are not quite sure what the reviewer is asking here, but have provided an explanation in any case. Both plates contain arabinose, and in both strains with pYhcB, the arabinose-induced expression of yhcB results in a ‘transparent’ colony morphology. However, as can be observed by both the spot count, and the growth in broth followed by microscopy, the change in transparency does not necessarily indicate toxicity. Given that these cells are viable, we suspect that the change in transparency is caused by an alteration (as yet unknown) at the cell surface. Anecdotally, we have also observed that leaky overexpression of pLpxC also results in a transparent cell morphology on spot plates without loss of viability, and wonder if changes in the surface LPS account for this phenomenon.

*e) How do the authors explain 2 different cell morphology phenotypes for the mutant grown in M9? Is this due to an accumulation of additional mutations in the population?*

Our hypothesis for this variation is related to the function of YhcB. Following growth in LB, we observed a wide variation in non-uniform cell lengths, suggesting a degree of stochasticity involved with cell division and loss of correct Z-ring timing. While growth in nutrient limiting conditions reduces phospholipid synthesis and cell size, and is somewhat restorative to a *yhcB* mutant, the underlying defect caused by *yhcB*-deletion is still present.

*2) Dissection of YhcB functional domains.*

*a) What is EV in Fig 4C – I presume it is empty vector?*

We apologise, this has been amended in the figure legend.

*b) Line 205-206 – the text suggests Fig 4A offers data on the functional importance of the PRDY data; it does not, it only shows conservation.*

We apologise but are confused by the referees point. (Former) Figure 4A does not show conservation data, it shows the transposon insertion sites along *yhcB* (of the original BW25113 transposon library following exposure to polymyxin B). The peaks represent the insertion loci of mutants that were able to grow when exposed to polymyxin B. Underneath the gene track are boxes representing specific domains and motifs within YhcB (to scale). This figure indicates that Tn-disruption to the ‘PRDY’ region does not affect cell growth in the presence of polymyxin B. In contrast, Figure S4B shows the amino acid conservation, as taken from across 150 different species. The widespread amino acid conservation across the full range of species would indicate structural or functional importance.

*c) The data points to the critical part of YhcB being cytoplasmic. How does this connect to the locations of other proteins identified by the screen, including “components of the elongasome” (line 264).*

This is presented in the schematic of Figure 11. With the exception of MurG, all proteins shown to interact with YhcB by BACTH (RodZ, MreD, MreC, RodA; Li et al. 2012) have TM domains. MurG is cytosolic, and RodZ has a cytoplasmic domain. Data presented by Li et al. (2012) suggest the TM domains of RodZ and YhcB are most critical for interaction.

Overall our hypothesis is that the cytoplasmic domain of YhcB is required for function and the TM domain is required for anchoring in the membrane for localisation. We believe that the truncated TM construct is able to complement loss of the full length protein because the functional portion of the protein is still present. We hypothesise that induced expression of the truncated construct results in a higher number of proteins, which can potentially overcome the loss of targeted localisation.

*3) RodZ essentiality.*

*a) Please explain how insertions in the 5-prime end of the gene are not polar on expression of the 3-prime essential region.*

We are unsure of the referees point here. *rodZ* has been presented in the orientation it is annotated in the reference genome, with the 5’ end of the gene on the right-hand side of the page, therefore the insertions observed in the diagram are in the 3’ not in the 5’ end of the gene. The *rodZ* gene is portrayed by a directional arrow, we have added annotations to the N- and C-terminus for additional clarity.

*b) please describe the topology of RodZ to explain how it can interact with the cytoplasmic part of YhcB; this might not be obvious to a non-specialist.*

We have added the text “RodZ is a bitopic CM protein that couples peptidoglycan synthesis to the MreB cytoskeleton” and included additional labelling in Fig. 5C for clarity.

*b) the Discussion (line 555) states that loss of the elongasome is restorative. Do the authors mean complete loss of elongasome activity or substantial reduction in elongasome activity?*

We thank the reviewer for highlighting this discrepancy and have rephrased this to specify loss of PG synthesis mediated by lpoA/mrcA rather than loss of the ‘elongasome’ in toto, as the core components are still essential. “our data reveal that loss of PG synthesis by PBP1A in the elongasome is restorative.”

*4) Suppressors of yhcB mutant defects.*

*a) The observation that insertions upstream of uppS and cdsA are suppressors is very interesting. To confirm the later interpretations, it would be helpful to provide some additional confirmatory insight i.e. do these mutants lead to altered transcription of the locus?*

We shared the same intrigue on this point and have explored it further by evaluating expression of the *uppS* and *cdsA* genes by RT-PCR. In addition to testing the effect of an antisense-*cdsA* construct. This data, coupled with the existing *uppS/ cdsA* overexpression data has been collated into it’s own figure. There is, however, much more to be understood about the regulation of such a highly conserved and functionally important operon.

*b) Some additional data relevant to ECA and UppS appears in the last section of the results and I think it would be clearer if this were unified with the earlier discussion. Otherwise it seems a bit repetitive.*

We thank the referee for their thoughts. We have decided to keep the section on UppS/CdsA in the same position and have added data to support the TIS data; demonstrating that knockdown of *cdsA* and overexpression of *uppS* had independent but restorative effects on the *yhcB* phenotype.

*5) The membrane ruffling visualized by simple TEM in Fig 10A and Fig S10 does not seem so convincing, considering the severe phenotypes. TEM does not seem the right approach here. The freeze substitution images in Figs 10B S11 and S12 are far more compelling. I suggest removing the TEM image from Fig 10 and deleting Fig S10 since these are mostly redundant.*

The simple TEM images have been relegated to supplementary material.

*6) Accumulation of LPE in the yhcB mutant. This seems an important observation and I believe it merits a more robust identification than that provided by TLC migration and correlated PagP activity. This should be amenable to MS given the abundance of the TLC spot. The text (line 440) refers to Fig 10B for LPE identification; it should be 10C.*

This has been added to the manuscript.

**Other editorial points:**

*Where YhcB is first mentioned in the Introduction, it would be helpful to include the few references specific to this protein (e.g. doi: 10.1016/j.jbc.2021.100700; 10.1007/s12275-020-0078-4; 10.5402/2012/304021).*

We have added into the intro: “More recent studies have shown that loss of YhcB results in a more permeable cell envelope and increased sensitivity to cell wall-targeting antibiotics (Sung et al. 2020; Mehla et al. 2021).”

*The initial screen/selection was done with 0.2 micrograms/mL; what is the MIC for this strain?*

The range reported for mcr-1 negative *E. coli* isolates is 0.25-2 µg/ml (<https://journals.asm.org/doi/full/10.1128/JCM.00268-17> ; <https://file.qums.ac.ir/repository/mmrc/clsi%202017.pdf> ). We did not determine a clinical ‘minimum inhibitory concentration’ in the strictest sense of the term, as defined by Andrews ([10.1093/jac/48.suppl_1.5](https://doi.org/10.1093/jac/48.suppl_1.5)) for this strain. In part because Polymyxins are notoriously challenging to define an inhibitory concentration for, as discussed here (<https://www.ncbi.nlm.nih.gov/pmc/articles/PMC6440778/> ). The ‘MIC’ varies with medium used and cell density. In particular, our work required the use of LB medium which is not cation controlled, a known confounding factor when defining an MIC for polymyxins. When we tested conditions to reflect the conditions of a TraDIS screen (using a glass flask with 50 ml LB, aeration, and a higher inoculum than typical MIC assays), we identified an MIC in the range of 0.5-1 µg/ml. Both ICs are within the range reported for mcr-1 negative *E. coli* isolates, and the concentration of 0.2 µg/ml polymyxin B is below the ICs. Therefore, we considered 0.2 µg/ml polymyxin B to be sub-inhibitory under the conditions tested, but hesitate to quantify this concentration as a measure of MIC for the reasons outlined above.

*Line 301: I am not sure “LPS stability” is the right term here.*

Thank you. This has been updated in the text and replaced with “OM rigidity or structural integrity”

*Line 313: this references relevant work from Young’s group on sequestration of undecaprenyl phosphate in ECA defects. A recent paper from Troutman’s group should also be added (doi: 10.1021/acschembio.0c00983).*

This reference has been added to the discussion

*Line 466: the reference to Fig S11 is incorrect.*

This has been corrected

*How many replicates were done for the PG analysis?*

Three, the data for which are included in Supplementary Table 5.

*Line 575-577: How does conservation of uppS-cdsA reveal a tight linkage between PL production and polysaccharide biosynthesis*.

We replaced polysaccharide with peptidoglycan.
