# Supplementary material for: Loss of YhcB results in dysregulation of coordinated peptidoglycan, LPS and phospholipid synthesis during Escherichia coli cell growth
Source: PLoS Genet. 2021 Dec 23;17(12):e1009586. doi: 10.1371/journal.pgen.1009586 (PMC8741058; doi:10.1371/journal.pgen.1009586)
Supplement: Attachment — Submitted filename: 211118_YhcB reviewers comments 2.0.docx [file pgen.1009586.s033.docx]

YhcB reviewers comments #2

There are still some issues:
1) The main conclusion, as highlighted in the title, abstract, and throughout the manuscript is that YhcB coordinates peptidoglycan and LPS biogenesis with phospholipid synthesis. In some cases, YhcB is referred to as regulator (e. g. lines 57-59, 651). The authors present evidence connecting YhcB to these processes through phenotypes and/or genetic connections. However, they do not provide evidence that YhcB coordinates these processes. As the authors state, growing cells coordinate peptidoglycan, LPS, and phospholipid biogenesis. Therefore, a factor that affects all three processes could be acting as a true coordinating factor of two or more of these processes or be a factor that directly affects only one of them and indirectly affects the other two because of the inherent coordination that exists in the cell. In fact, the authors state (lines 550-553) that the “loss of yhcB results in an increased demand for the Und-PP synthase, resulting in upregulation of the uppS-cdsA operon. Increased expression of cdsA in turn gives rise to excess phospholipid synthesis, resulting in detergent sensitivity and enlarged cells.” In other words, the authors explain that the phenotypes they observe in the yhcB mutant result from an unexplained deficiency of Und-PP that ends up upregulating phospholipid synthesis simply because a gene for phospholipid synthesis (cdsA) is in an operon with uppS, which encodes for a Und-PP synthase. Furthermore, the connections to LPS biogenesis appears to come from genetic interactions that ultimately decrease phospholipid biosynthesis by increasing LPS synthesis through their balanced synthesis. This study provides a lot on information on YhcB but we still do not really know its function and what the primary defect is resulting from its loss. The authors provide a reasonable explanation supporting an indirect effect on phospholipid synthesis, which ultimately also links it to LPS biogenesis. Therefore, the authors need to change their title and assertions that YhcB coordinates peptidoglycan, LPS, and phospholipid biogenesis. The running title should also be edited.

In response to the referees comments we have altered the title to read **Loss of YhcB results in dysregulation of coordinated peptidoglycan, LPS and phospholipid synthesis during *Escherichia coli* cell growth.** The running title was altered to **Loss of YhcB results in dysregulated cell envelope synthesis.**

2) Readers will have to commit a significant amount of time to read and go over the data presented in this manuscript. Given the type and amount of data in the paper, I think that it would be extremely helpful to readers if key data from some of the supplementary tables that the authors highlight in the figures and text is presented in an easily accessible form. This is specifically relevant to Tables S3, S4, and S6. For example, the excel file for Table S3 could have an additional sheet displaying the list of the 28 genes highlighted in the text and Fig 4B as being essential in the wild type and not in the yhcB mutant. The same approach could be used to report the list of genes contributing to fitness reported in Table S4. Otherwise, readers need to sort through the information, and some readers might not have the knowledge to do it. In fact, I spent quite a bit of time doing this, and my numbers do not match those reported by the authors. It is possible that I made mistakes or was not given all the information I needed, reinforcing the importance of providing a concise list of the relevant data (in addition to the complete data), especially to understand why the authors focused on select data. After sorting the table S3 using the authors’ annotations of "essential" and "non-essential", I only found 16 genes, not 28, as essential in the wild type but not the mutant, and 108, instead of 121, as essential in the yhcB strain but not the wild type. The numbers I obtained from Table S4 do not match either; likely, I obtained larger numbers because the authors filtered out essential genes when reporting the relevant data and I did not because that information is not provided in Table S4. For Table S6 (and text in line 365), it is unclear which of the suppressors listed in Table S6 are the 28 "universal suppressors” since cross-referencing both lists yields 43 in common (40 genes and 3 intergenic regions).

We thank the referee for doing their own analysis but note that their analysis of our data was flawed. However, we have amended the manuscript to minimise misunderstanding, it now reads “We identified 28 knockout suppressor mutations shared between both conditions”. We have made the Tables clearer where necessary.

3) On the analysis of the different domains of YhcB: The authors never tested the mutant that only lacks the helical domain, so it's a bit of a stretch to state that the “data suggest a dual function of the YhcB protein: the cytoplasmic helical domain is needed to suppress sensitivity to SDS and EDTA, while the function of the ‘PRDY’ domain is critical for survival in the presence of vancomycin, but not polymyxin B or SDS and EDTA” (lines 234-237). More importantly, these data do not necessarily indicate a dual function. At the very least, having two functions is not the only explanation for their results. What the authors are reporting could result from a gradient of defects in one single function. It seems that some mutants can grow on SDS/EDTA plates but not on vancomycin plates. Also, slow growth restores growth on SDS/EDTA but not vancomycin. One possible explanation for all these observations is that vancomycin plates, at the concentration used, demand more of the function that is defective in yhcB mutants than SDS/EDTA plates at the concentration used. In fact, the authors state that to "confirm the barrier defect of the yhcB mutant, we grew the parent strain, E. coli BW25113, and our isogenic DyhcB mutant on LB agar plates supplemented with either vancomycin, a high molecular weight antibiotic that is typically unable to cross the Gram-negative OM, or plates supplemented with SDS and EDTA" (lines 163-166). Since sensitivity to vancomycin, SDS/EDTA, and polymyxin result from a "barrier defect", the statement in lines 163-166 seems to be at odds with using SDS/EDTA and vancomycin to discriminate between different functions. If the authors want to propose two functions for YhcB based on differential sensitivity of the domain mutants, they need to explain their reasoning in a way that does not disagree with their explanations for how their chemical selections and phenotypic analyses work.

We thank the referee for highlighting this point. To be clear

1. YhcB physically interacts with components from different cell envelope assembly pathways.
2. Deletion of different domains results in different chemical sensitivities
3. We have demonstrated through genetic screens that compensatory mutants can give rise to differential restoration of barrier functions.
4. We have demonstrated that the restoration of the barrier function and cell size can be separated by different compensatory mutations.

Collectively, this indicates a dual role for YhcB. However, we have adjusted the text to read: “**Further work is needed to characterise the role of the PRDY domain,** however these data suggest the function of the ‘PRDY’ domain is **condition-specific** as it is critical for survival in the presence of vancomycin, but not polymyxin B or SDS and EDTA. **Given the highly conserved nature of this domain across multiple species, it is our hypothesis that this domain is functionally important and is functionally distinct from the cytoplasmic helical domain.”**

4) The authors stated that the mla* allele is toxic in the wild type but not in the yhcB mutant. I assume this refers to the stationary-phase death reported for the mla* mutant by others. The authors showed that mlaA* suppresses yhcB, but I do not think they showed that the stationary-phase death in the mla* mutant is corrected in the yhcB mutant. It might be true, but it is not known unless I missed it. These data are not required, the authors can just remove their claim (might be in a couple of places).

We have rephrased the text: **the *mlaA** mutation is restorative** in a Δ*yhcB* mutant, restoring both cell size and resistance to vancomycin.

5) The authors did not remove the sentence "With thousands of deleterious and restorative genetic interactions discovered, to the best of our knowledge no other gene has been scrutinised genetically at such scale and resolution" (lines 557-559) because they “believe it to be true”. It is disappointing and quite telling that they feel the need to keep an unnecessary statement that can only be substantiated with their “belief”. Moreover, "thousands" of genetic interactions must imply that they are counting every transposon insertion as one interaction. So, if a gene has 100 different insertions and all of them simply result in functionally equivalent null alleles, the authors are counting them as 100 genetic interactions. Although technically correct, it is an interesting choice of words to describe their work. As a reviewer, I appreciate that the authors chose to write the manuscript at the gene level, not the insertion level.

We thank the referee for recognising we are correct. Nevertheless, we adjusted the text to read “With thousands of deleterious and restorative genetic interactions discovered, **TIS is a powerful tool that can transform our understanding of poorly characterised genes**.”

6) The authors’ attempt to fix the sentence regarding counting and enumerating the parts on the cell envelope is still problematic. The revised sentence reads: “In Gram-negative bacteria, the tripartite cell envelope is composed of the cytoplasmic membrane (CM), the peptidoglycan (PG) layer within the periplasmic space and an outer membrane (OM)” (lines 66-68). This sentence is still incorrect. “Tripartite” indicates 3 parts but there are 4: CM, PG layer, periplasmic space, and OM. Based on the original and revised sentences, it appears that the authors do not recognize the periplasm as a part of the cell envelope. This is quite surprising and makes me wonder if they consider the cytoplasm a part of the cell.

Noted.

7) In response to the authors’ answer about the question of whether suppressors are specific to the loss of yhcB (response to point 4 by reviewer 1): Their answer provided is partly confusing. Their argument about the mla suppressors makes sense since the single mutants are sensitive but the double mutants are more resistant to SDS/EDTA. However, the rest of the answer is irrelevant to the point of the question. It remains to be known how specific other suppressors are. I'm not asking for the authors to test them but to acknowledge this issue in their text. I hope the authors also recognize that the validation of "some of their findings" does not give a blanket validation of all other mutants as they imply in their answer.

We have added the following sentence for clarity

“**Targeted validation experiments would be needed to distinguish specific from non-specific suppressor mutations, however the collated TIS data can highlight functions that are restorative in a *yhcB* mutant for further investigation**.”

8) Authors’ response to Reviewer 1, point 9: although the change is acceptable, I want to clarify the reason for my previous request since the authors were not sure. Your study used transposon mutagenesis to generate mutants and reports on a few spontaneous suppressors. The sentence was an absolute statement about having found "all" genes that suppress the yhcB null. The old sentence was incorrect because 1) only the transposon mutant collection was saturated in this study and there are other types of alleles that might suppress that could never be generated by transposons, and 2) there is no indication that the spontaneous selections were saturated.

We thank the reviewer for clarifying their earlier comments.

9) Lines 56-57: “YhcB influences the temporal biogenesis of LPS, peptidoglycan and membrane phospholipids.” I am not sure why the authors specify a “temporal” effect as opposed to, for example, an effect on the balance of their synthesis (amount). If the temporal reference relates to an excess phospholipid synthesis occurring in stationary phase, then it would be more appropriate to refer to the physiological state of the cell (not time) but would raise questions about YhcB’s role during exponential growth.

We thank for reviewer for noting this, this was an oversight in the initial submission and has been corrected to: “Subsequent molecular and biochemical analyses suggest YhcB influences the spatiotemporal biogenesis of LPS, peptidoglycan and membrane phospholipids.”

10) Lines 159-160: “We observed that the ΔyhcB mutants from the Keio library do not phenocopy each other.” The new statement is confusing. Is it that there is more than one *yhcB* mutant in the collection, or that different colonies from one single mutant behaved differently, or that the same mutant from different copies of the collection in different labs do not behave the same? In addition, in the Methods section, it seems that the authors constructed a new *yhcB* mutant by moving the allele from their Keio collection into their wild type by transduction based on their description. Given that they raise concerns about the mutant in their Keio collection, it might be useful to readers if they clarified that if allele in the Keio collection is correct and can be used if moved into a new strain.

We have updated the manuscript to read

“We observed that the two D*yhcB* mutants from the Keio library do not phenocopy each other. Whole genome sequencing revealed mutations at secondary sites in both isolates when compared to the parent strain, *E. coli* BW25113”

All of the raw sequencing data have already been made publicly available, however, to aid readers we have added more details to Table S11 with the footnote that we have not tested other Keio libraries.

11) Line 364: given that mutants were generated by transposition, referring to gene-deletion suppressor mutations is incorrect.

This is a subtle point to address and resolve. We have updated the text to read “We identified 28 “knockout” suppressor mutations shared between both conditions, and therefore considered these to be universal suppressors”, but please see below for the justification.

We are in agreement with the reviewer that a single mutant containing a single transposon insertion within a single gene would be classified as a “gene-disruption” mutant. However, in this context we are referring to observations made of multiple mutants in tandem. The naming convention used here is in keeping with the format presented by Page et al. (2020) in their publication of AlbaTraDIS. A feature of the AlbaTraDIS package is the prediction of the type of suppression derived from the insertion data pattern, (listed and defined in the AlbaTraDIS publication). For example, genes with multiple transposon insertion events recorded along the length of the protein coding sequence of the gene is characteristic of suppression tantamount to gene deletion. As this was the TIS analysis method we applied, the language has previously been defined by Page et al. (2020), and we observed the same patterns within our data for the majority of the 28 genes, we chose to maintain this standard.

However, it is important to stress that predictive analysis is not definitive evidence, and that the classification of biological data into simplified outputs will inevitably require caveats. In the select few genes where the insertion pattern does not conform to the expected insertion pattern for a knockout suppressor mutant, we have now added a footnote to supplementary table 6.

12) Line 590: Rephrase "mutations within the metal ion binding site of CdsA" since mutations occur in DNA, and the relevant amino acids are located near but not within the metal-binding site.

This has been changed to “**amino acid alterations** **proximal** to the metal ion binding site of CdsA”. But please note, for one of the residues (S239) the analogous residue in CdsA of *T. maritoma* (S223), for which the crystal structure has been solved, *does* coordinate metal ion co-factors.

13) Lines 595 and 602: Please specify (to clarify to readers) that the listed findings pertain to the *yhcB* mutant. Done.

14) Line 614: “it’s” should be “its” Corrected.

15) Table S1 and lines 148-149: yjbS was not in category "S" even though is defined as "uncharacterized protein" in Table S1?

The definitions listed in Table S1 are extracted from the general feature format (.gff) annotation data downloaded from NCBI, while the COG categories were assigned by eggNOG-Mapper (<http://eggnog-mapper.embl.de/>). yjbS was not assigned a COG category by eggNOG-Mapper. This can happen when a gene has insufficient orthologs at the time of analysis to be assigned a ‘Cluster of Orthologous Groups’ category. (Please note, COG analysis was repeated 19^th^ March 2021 prior to submission to account for database updates prior to publication).

The text presented in the manuscript is therefore accurate, but we have updated it to reflect the date accessed.

“Comparison of the relative abundance of COG (Cluster of Orthologous Groups) categories of these 54 genes showed a marked increase in genes involved in cell wall/membrane/envelope biogenesis (COG category ‘M’), supporting the validity of the TIS screen (Fig. S1D; Huertas-Cepas et al. 2019; <http://eggnog-mapper.embl.de/> date accessed: 19^th^ March 2021). … The COG analysis identified only three genes (*ydbH*, *yqjA* and *yhcB*) of unknown function (category 'S' in Fig. S1D) that were important for survival when exposed to sub-inhibitory concentrations of polymyxin B (Fig. 1C and Fig. S1E).”

It would be accurate to add: “There was one gene, *yjbS*, that was not assigned a COG category, but is annotated as an ‘uncharacterized protein’ in the reference genome, that was also identified as important for survival in polymyxin B.”
On consideration, we find this addition to be disruptive to the flow of the manuscript and have not included this sentence but would be happy to make the change if requested by the editor.

16) Table S7: in the excel file, it is mislabeled as Table S8. Also, what is a “conservative in-frame” deletion/insertion? One that conserves the reading frame? Isn’t that denoted by “in-frame”?

We have deleted the word ‘conservative’ and corrected ‘8’ to ‘7’ within the document, we thank the reviewer for highlighting this.

17) It would also be helpful if the authors kindly helped readers by adding the relevant genotypes to the labels in Fig. S9 instead of only using “mut#” labels.

A simplified genotype has been included in the figure legend.

We are deeply thankful to reviewer 1 for the time invested in improving this manuscript.

Reviewer #2: The authors have given careful consideration to my comments and I am satisfied with the changes and response to comments. One minor point the authors might consider is to briefly comment on the crystal structure in the "domains of YhcB..." section to offer more context.

We would like to take this opportunity to thank reviewer 2 for their time and attention in reviewing this manuscript and their helpful suggestions for improving the work.

We have made the following brief addition “**A recent crystal structure was resolved for the TM and cytoplasmic helical domains and corroborates the multiple lines of evidence that YhcB self-interacts (Maddalo et al. 2011; Li et al. 2012; Mehla et al. 2021).”**
